# Dual Inhibitors of Acetylcholinesterase and Monoamine Oxidase-B for the Treatment of Alzheimer’s Disease

**DOI:** 10.3390/molecules30142975

**Published:** 2025-07-15

**Authors:** Ayesha Asim, Michał K. Jastrzębski, Agnieszka A. Kaczor

**Affiliations:** 1 Department of Synthesis and Chemical Technology of Pharmaceutical Substances with Computer Modeling Laboratory, Faculty of Pharmacy, Medical University of Lublin, 4A Chodźki St., PL-20093 Lublin, Poland; michal.jastrz1998@gmail.com; 2 School of Pharmacy, University of Eastern Finland, Yliopistonranta 1, P.O. Box 1627, FI-70211 Kuopio, Finland

**Keywords:** dual inhibitors, Acetylcholine Esterase (AChE), Monoamine Oxidase-B (MAO-B), molecular modeling, SAR

## Abstract

Alzheimer’s disease (AD) is a multi-factorial neurodegenerative disease with a complex pathomechanism that can be best treated with multi-target medications. Among the possible molecular targets involved in AD, acetylcholinesterase (AChE) and monoamine oxidase B (MAO-B) are well recognized because they control the neurotransmitters responsible for memory processes. This review discusses the current understanding of AD pathology, recent advances in AD treatment, and recent reports in the field of dual AChE/MAO-B inhibitors for treating AD. We provide a classification of dual inhibitors based on their chemical structure and describe active compounds belonging to, i.a., chalcones, coumarins, chromones, imines, and hydrazones. Special emphasis is given to the computer-aided strategies of dual inhibitors design, their structure–activity relationships, and their interactions with the molecular targets at the molecular level.

## 1. Introduction

### 1.1. Alzheimer’s Disease

AD is a neurological condition that develops over time and has a complex etiology. AD is the most common form of dementia, accounting for 60–70% of cases, affecting over 50 million individuals globally. It is estimated that there will be 82 million AD patients overall by 2030, and 152 million people will be impacted by the condition by 2050 [1]. The AD brain is characterized microscopically by the combined presence of two classes of abnormal structures, namely, extracellular amyloid plaques (Aβ) and intraneuronal neurofibrillary tangles (NFTs), both of which comprise highly insoluble, densely packed filaments. It leads to a progressive decline in cognitive function and memory loss.

The hallmarks of AD are generally considered to be the progressive neurodegeneration brought on by the death of neurons in the hippocampus and adjacent parahippocampal region of the brain, as well as the abnormal build-up of amyloidogenic proteins or τ protein in the affected brain areas [2]. There are two types of AD, which are etiologically and epidemiologically distinct: early-onset AD (EOAD) and late-onset AD (LOAD). LOAD, the most common form of AD, with a complicated etiology that accounts for approximately 95% of cases, is more common than EOAD [3]. EOAD, sometimes referred to as familial AD (FAD), can have a non-dominant etiology, such as mutations in the ε4 form of the apolipoprotein E (APOE4) allele, and can be inherited in an autosomal dominant way [4]. Five million people in the USA alone suffer from LOAD, a sporadic AD (SAD) form, which is the most prevalent age-related neurodegenerative disease caused by a variety of (mostly environmental) causes [5]. Numerous experimental, biochemical, neuropathological, and genetic studies have produced compelling evidence that the pathobiology of AD is ascribed to a variety of factors, giving rise to a multitude of theories and hypotheses regarding the various processes of AD pathogenesis. However, the most important risk factor for AD is thought to be aging.

### 1.2. Proposed Pathomechanism of AD

**Figure 1:**
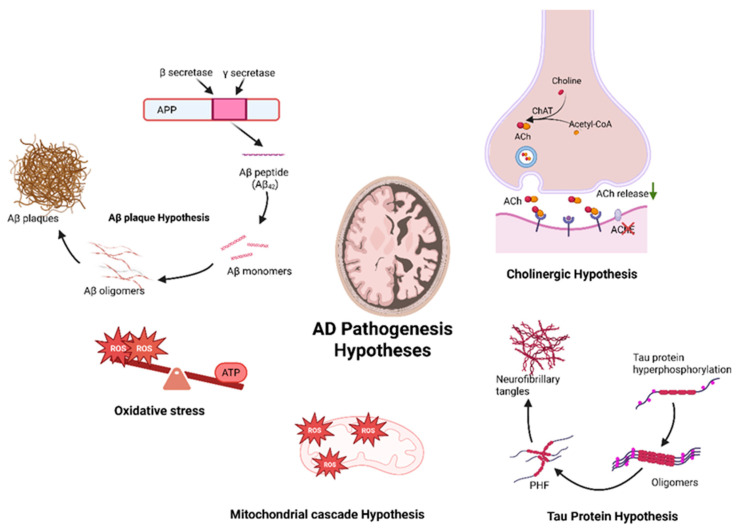
Schematic illustration of multiple pathogenesis hypotheses implicated in Alzheimer’s disease, including the following: (1) Aβ plaque hypothesis, characterized by the accumulation of Aβ peptides leading to the build-up of plaques; (2) cholinergic hypothesis, proposing deficiency of ACh neurotransmitter in the synaptic cleft; (3) the tau hypothesis, where hyperphosphorylation of tau proteins results in the formation of NFTs; (4) the mitochondrial cascade hypothesis, based on the mitochondrial dysfunction; and (5) oxidative stress hypothesis, proposing a high concentration of ROS and energy imbalance in the system as an early hallmark of AD progression.

Several mechanisms have been described to explain the pathology of AD [3,6]. Some of the proposed mechanisms of AD include the τ hypothesis, the amyloid cascade hypothesis, excitotoxicity, cholinergic hypothesis, and other concepts [7]. An overview of the major AD hypotheses is presented in Figure 1.

#### 1.2.1. The Cholinergic Hypothesis

The cholinergic hypothesis of AD describes a disease pathomechanism that takes into account a cholinergic deficit arising from a degenerative process damaging neurons in the hippocampus, frontal cortex, amygdala, basal nucleus, and medial septum—regions and structures with important functional roles in conscious awareness, affecting mnemonic processes in the central nervous system (CNS) [8,9]. The hypothesis takes into account three concepts: decreased levels of cholinergic markers in the cortex, effects on neurodegeneration in the forebrain affecting cholinergic innervation, and the role of cholinergic antagonists on memory [10].

In Alzheimer’s disease, amyloid-β (Aβ) disrupts cholinergic neurotransmission by impairing acetylcholine synthesis and vesicular transport. This results in reduced expression of cholinergic markers, including choline acetyltransferase (ChAT) and vesicular acetylcholine transporter (VAChT), contributing to cognitive deficits characteristic of the disease (Figure 1). A deficit in essential amino acids and in nicotinic and muscarinic receptors neurotransmission has also been noted [10,11].

An important aspect is the relationship between the cholinergic and glutamatergic systems during neurotransmission changes. Excessive depolarization of the postsynaptic membrane as a result of the hyperactivation of *N*-methyl-D-aspartate (NMDA) glutamatergic receptors results in excessively high levels of calcium and sodium ions, which contributes to neurodegenerative processes [12,13] and can generate long-term depression (LTD) in the cerebellum. It is also associated with calcium overload in mitochondria, free radical generation, or initiation of apoptosis [14,15].

The cholinergic hypothesis has served as the basis for most treatment strategies and drug development approaches (acetylcholinesterase inhibitors, cholinergic precursors, cholinergic receptor agonists, allosteric cholinergic receptor enhancers) for AD. According to our current knowledge, the association between cognitive impairment and reduced cholinergic transmission in the brain plays an important role in AD but does not by itself establish a definitive cause of the disease [9].

#### 1.2.2. The Amyloid Hypothesis

Aβ is a 40- or 42-amino-acid peptide derived from the amyloid precursor protein (APP), a transmembrane protein, through sequential cleavage by β-secretase (BACE1) and γ-secretase. The amyloid cascade hypothesis initially posited that the accumulation and deposition of amyloid-β (Aβ) peptide and the formation of amyloid plaques in the central nervous system were strongly correlated with dementia and played a key role in the pathogenesis of AD (Figure 1). However, subsequent research has shown that amyloid plaques can also be found in the brains of cognitively normal older individuals, and that plaque burden does not always correlate with neuronal loss or cognitive decline. Consequently, the hypothesis has evolved to emphasize that it is the soluble oligomeric forms of Aβ, not the insoluble plaque deposits, that are primarily responsible for driving the disease process [9].

AD brain cortical plaques are mainly composed of Aβ protein. Abnormal cleavage of APP protein results in the formation of Aβ. The specific physiological roles of APP are not entirely clear, but it is generally thought to contribute to normal neuronal function and brain development. The amyloid hypothesis suggests that cleavage of amyloid precursor protein (APP) by β-secretase and γ-secretase leads to the production of amyloid-β (Aβ) peptides, particularly Aβ40 and the more aggregation-prone Aβ42. Impaired clearance or increased production of Aβ, especially under aging or pathological conditions, leads to its accumulation and plaque formation, contributing to Alzheimer’s disease pathology. An increase in the Aβ42/Aβ40 ratio induces the formation of Aβ amyloid fibrils, resulting in neurotoxicity and the induction of τ pathology, which ultimately leads to neuronal cell death and neurodegeneration [16].

The processing of the amyloid precursor protein (APP) occurs via two major pathways involving different secretases. In the non-amyloidogenic pathway, APP is initially cleaved by α-secretase enzymes, such as ADAM family metalloproteinases (e.g., ADAM10), leading to the release of a soluble *N*-terminal fragment called APPsα, which has neuroprotective functions and is found at reduced levels in Alzheimer’s disease (AD) [17]. In contrast, the amyloidogenic pathway begins with cleavage by β-secretase (BACE1), followed by γ-secretase, resulting in the production of amyloid-β peptides. BACE1 expression may be upregulated under conditions commonly associated with neurodegenerative diseases and aging [9,18].

The translocation of amyloid-β across the blood–brain barrier (BBB) via the receptor for advanced glycation end products (RAGE) plays a significant role in AD. The interaction between the RAGE and Aβ can trigger inflammatory responses at the endothelial level and induce apoptosis of endothelial cells, contributing to the neurovascular dysfunction observed in AD [9,19].

The amyloid cascade hypothesis has a high acceptance rate; however, current research supports the view that it is not only amyloid β that contributes to AD pathogenesis.

#### 1.2.3. The Tau (τ) Protein Hypothesis

τ is a highly soluble microtubule-associated protein that enhances the polymerization and stabilization of microtubules in the cell cytoskeleton [9]. The stabilization of microtubules promotes structural change functions, axonal transport, and neuronal growth [9]. A large accumulation of τ protein in the hippocampus is characteristic of a subtype of AD called the “limbic-predominant type.” When the accumulation of τ in the hippocampus is milder than expected in light of the pathology, this is referred to as “hippocampal sparing” [20].

In the τ hypothesis, it is assumed that the hyperphosphorylated τ protein, which arises as a secondary pathogenic event, is displaced from microtubules and aggregates into paired helical filaments (PHFs) and NFTs, resulting in neurodegeneration before plaque A formation (Figure 1). The Apo epsilon 4 (APoe4) allele of the apolipoprotein (apo) gene encoding a protein involved in cholesterol metabolism and lipid transport is thought to play an important role in this process [6,11].

The τ hypothesis is in part an extension of the amyloid cascade hypothesis; in vitro experiments using neuronal cell lines, primary hippocampal and cortical neurons, and organotypic cultures of the hippocampus have shown that amyloid β induces τ changes [21,22]. According to the available literature, changes in τ protein and amyloid β oligomers are the most important factors responsible for neuronal dysfunction in AD pathogenesis [23]. The description of τ pathology correlates better with cognitive impairment than Aβ damage, but there is no uniform theory to justify its important role [24].

#### 1.2.4. The Mitochondrial Cascade Hypothesis

The mitochondrial cascade hypothesis assumes that similar physiological mechanisms underlie AD and brain aging, and that AD mitochondrial dysfunction is not just a consequence of neurodegeneration. It is also postulated that mitochondrial dysfunction drives amyloidosis, τ phosphorylation, and re-entry into the cell cycle. The mitochondrial cascade hypothesis is an extension of the free radical theory of aging, using information on the role of somatic mitochondrial DNA (mtDNA) damage. The theory states that various mechanisms, including oxidative stress and proteasome dysfunction, facilitate mitochondrial dysfunction in neurodegenerative diseases such as AD [25].

AD patients exhibit reduced cytochrome c oxidase (Complex IV) activity in platelets, which contain mitochondria and mitochondrial DNA (mtDNA). Cybrid cell models incorporating mitochondria from AD patients also show diminished cytochrome oxidase activity and produce excess Aβ40 and Aβ42. These findings support a link between mitochondrial dysfunction and amyloidogenesis [26]. Dysfunction of the mitochondrial electron transport chain (ETC) increases reactive oxygen species (ROS) production, contributing to oxidative stress. Moreover, human NT2 neuron-like cells exposed to Aβ peptides exhibit increased cell death, suggesting that mitochondrial ETC dysfunction mediates Aβ-induced neurotoxicity. Furthermore, APP, Aβ, and the entire γ-secretase complex are found in mitochondria or mitochondrial membranes [27].

There is also a hypothesis that one of the physiological roles of APP or Aβ may be to “turn off” abnormal mitochondria. If the altered physiology of APP or Aβ inadvertently induces mitochondrial dysfunction, this would trigger the same series of events as aging.

#### 1.2.5. Other Theories

Essential biometals, including zinc, copper, and iron, play a structural and catalytic role in several processes such as neurotransmitter synthesis and enzyme functioning. However, an imbalance of these metals leads to ROS production in the brain with Alzheimer’s disease [28]. The dyshomeostasis of Zn, Cu and Fe in the localized regions of the brain has been largely associated to Aβ aggregation. Particularly, redox metals, such as Cu and Fe, generate highly reactive hydroxyl radicals through Fenton reactions that ultimately contribute to DNA damage, protein oxidation, and mitochondrial dysfunction. In addition, Cu and Zn promote regional oxidative stress and chronic inflammation by binding to cytotoxic Aβ peptides and encouraging their aggregation. The metal-mediated reactive species along with MAO-B-mediated oxidative stress generate a cycle of neuronal injury in Alzheimer’s pathology [29]. Developing MTDL that possess metal chelating properties to restore the normal homeostasis of essential metals could be a promising approach for tackling the complex pathology of AD.

In 2010, it was shown that zinc release into the glutamatergic synapse, mediated by the ZnT3 transporter, affects memory and cognition [30]. ZnT3, found in grey matter regions affected by amyloid pathology, caused amyloid pathology in transgenic mice [31]. In AD, cognitive decline may result from amyloid trapping of extracellular zinc. Aging or disease disrupts metal homeostasis, causing aggregation, oxidative damage, and iron accumulation in neurons. Abnormal amyloid β accumulation has been shown to be capable of promoting ROS formation through a mechanism that involves the activation of NMDA receptors, the OS can increase amyloid β production and aggregation, as well as facilitate τ phosphorylation and polymerization [32]. Oxidative stress (OS), an imbalance between antioxidants and ROS, is linked to neuronal apoptosis in AD [33].

Moreover, the ghrelin receptor GHSR1α, mostly expressed in the hippocampus, influences learning and motivation by regulating dopamine D_1_ receptor (D_1_R) activity [34,35]. In non-pathological conditions, GHSR1α generates heterodimers with the D_1_ receptor to promote the normal function of the hippocampus. However, the binding of Aβ to GHSR1α induces hippocampal synaptic stress and memory deficits by preventing GHSR1α/D_1_R heterodimerization, affecting hippocampal function, and thereby impairing hippocampal-dependent cognitive processes [36].

From another perspective, microorganisms have also been suspected in AD pathogenesis. Evidence of inflammation in AD brain tissue, such as complement system activation and microglia response, suggests that Aβ plaques may serve a protective role against microbial threats in the brain of AD patients [37]. The concepts discussed above are summarized in Table 1.

## 2. Treatment of AD

### 2.1. Non-Pharmacological Treatment

According to WHO guidelines, reducing the risk of cognitive decline and dementia can be achieved by implementing recommendations on physical activity, diet, overweight or obesity, tobacco and alcohol use, hypertension, and diabetes [38,39].

Large-scale, long-term, randomized trials have investigated whether lifestyle changes can reduce the risk of cognitive impairment in at-risk individuals: the FINGER study (Finland) demonstrated the impact of nutrition, exercise, and social activity [40,41]; MAPT (France) investigated omega-3 fatty acid supplementation [42]; and the PreDIVA study (Netherlands) linked the improvement of AD patients to the pharmacological management of vascular and metabolic risk factors [43]. The results were inconclusive, but it is plausible that even if lifestyle changes do not affect the pathogenesis of AD, they may improve their clinical outcomes. Physical and mental exercise, involving event planning or logic games, are effective in the treatment of mild depression resulting from AD.

In the treatment of AD, attention should also be paid to the holistic aspect. Patients’ co-existing behavioral and non-neurological conditions must be optimally treated. The effects of the disease affect not only the person directly suffering from AD but also their families, so the involvement of companions is crucial to achieving the desired therapeutic outcome. AD itself and its associated conditions make it necessary to control cognitive impairment, depression, and dementia to help the patient manage money and time and move around freely.

A completely new non-pharmacological treatment method for AD includes the use of gamma oscillations. These alleviate the pathology and cognitive impairment in AD. Gamma oscillations are rhythmic brainwave fluctuations caused by the activation of local circuits of excitatory and fast-onset inhibitory neurons in the local field potential (LFP), resonating at 20–50 Hz and associated with numerous higher-order cognitive functions such as memory formation and attention selection [44]. Alterations in gamma oscillations have been noted in AD [45,46], as well as in the spatial effects in a mouse model of τ pathology [47]. Learning and memory deficits in mice with the human APOE4 gene were ameliorated using gamma oscillations [48]. By studying multiple mouse models using optogenetic methods in the hippocampus of 5XFAD mice, it was found that oscillations of light at 40 Hz reduce the level of AD pathology. The mechanism of this process is not clear, but GABAergic neurotransmission is assumed to be involved [6].

### 2.2. Pharmacological Treatment

**Figure 2:**
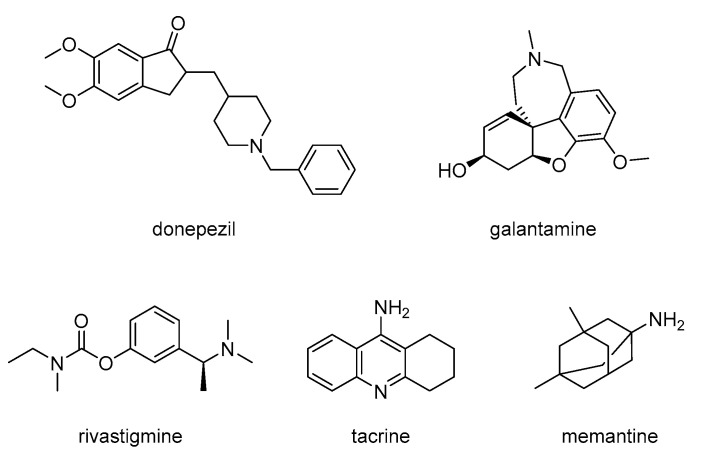
Structures of AChE inhibitors and NMDA antagonist used for treating AD.

Cholinesterase inhibitors (AChEIs) and NMDA receptor antagonists are well-established pharmacological treatments for AD. However, they can only alleviate symptoms and can slow down the progression of AD. Marketed AChE inhibitors include donepezil, galantamine, rivastigmine, and tacrine (though tacrine was withdrawn due to hepatotoxicity), while the NMDA antagonist used is memantine [8,49] (Figure 2).

#### 2.2.1. Acetylcholinesterase Inhibitors

Donepezil and galantamine are selective acetylcholinesterase inhibitors, while rivastigmine inhibits both acetylcholinesterase and butyrylcholinesterase, albeit with distinct affinities [50]. Galantamine also has allosteric effects on presynaptic nicotinic receptors [51].

Clinical studies and clinical practice have shown that the effects of cholinesterase inhibitors on cognition, memory, and concentration are moderate. These benefits are usually accompanied by a short-term stabilization of functional abilities. In addition, these inhibitors can cause moderate improvements in mood and social interaction and a reduction in apathetic states [52]. However, it is noteworthy that these effects are purely symptomatic and do not alter the underlying mechanism of progression of AD.

According to the available literature, drugs using the above mechanism can produce efficient long-term effects for up to 5 years [53]. Although inhibitors are effective in mild-to-moderate forms of the disease, one study indicates that donepezil is also effective in severe forms [54].

#### 2.2.2. Memantine

Glutamate (an excitatory neurotransmitter in the brain) and the NMDA receptor play an important role in learning and memory processes. This action is altered in AD, as increased glutamate activity leads to sustained, low-level activation of NMDA receptors, impairing neuronal function [11,55]. Memantine (Figure 2) is an uncompetitive NMDA receptor antagonist, protecting neurons from excitotoxicity without preventing the physiological activation of NMDA receptors, which is necessary for cognitive functioning [10,56]. A six-month course of memantine has been shown to improve the cognitive and behavioral symptoms in individuals with moderate-to-severe AD. The use of memantine after six months in patients with moderate-to-severe AD improves cognitive and behavioral symptoms [38], as well as the ability to carry out activities of daily living and has an effect on reducing agitation and the degree of aggression [57]. Combination therapy, which combines the use of acetylcholinesterase inhibitors and memantine has additional clinical benefits [18,58].

Classical AD treatments are attributed with limited efficacy and dose-dependent side effects. Cholinesterase inhibitors increase the occurrence of nausea, vomiting, and diarrhea [50]. Memantine, on the other hand, causes severe hypotension leading to fainting and falls [59].

#### 2.2.3. New Approaches to the Treatment of AD

Since current therapeutics of AD are based on symptomatic treatment only, there is a great need to search for disease-modifying therapies and to eliminate the side effects associated with typical cholinesterase inhibitors and NMDA antagonists.

Currently, there are more than 100 formulations in various phases of clinical trials. Novel AD therapies can be divided into two groups: therapies that modify the disease pathomechanism itself and therapies that provide symptomatic effects. AD therapies involve effects on the amyloid cascade (vaccines, antibodies, monoclonal, BACE β-secretase inhibitors) and τ protein (vaccines, monoclonal antibodies, microtubule stabilisers), among others (chelating agent, anti-inflammatory, metabolic, neuroprotective effects, gene therapy, stem cells) [60].

Metal chelation therapy has received significant attention due to the implication of metal dyshomeostasis in Aβ and oxidative stress. Metal chelating compounds, such as clioquinol and derivatives [61], 8-hydroxyquinoline derivatives, etc. [62], bind to the excess metal ions to cut ROS production and Aβ aggregation, thus reducing the plaque build-up and neurotoxicity. AN overview of classical pharmacological interventions and emerging technologies for AD is presented in Table 2.

**Figure 3:**
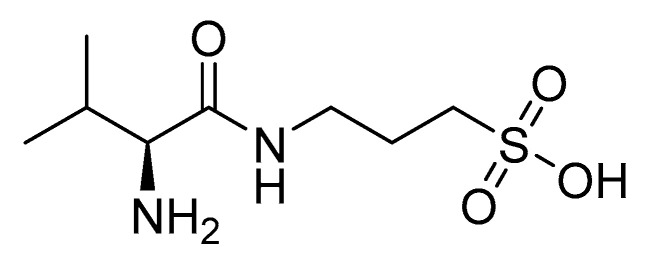
ALZ-801 (valiltramiprosate).

In recent years, several monoclonal antibodies targeting amyloid beta plaque have been developed and approved for the treatment of AD [63]. Monoclonal antibodies have proved to be the first disease-modifying treatment for AD. Aducanumab targeting Aβ was the first drug approved in 2021 to modify the course of AD treatment altogether. However, its use has been controversial and is still debated because of poor results on cognitive decline during clinical trials [64]. Another monoclonal antibody, lecanemab, that binds strongly to soluble Aβ protofibrils to inhibit aggregation in the early stage of AD, was fully approved by the FDA in 2023 [63]. Furthermore, donanemab showed promising results in phase-III trials in December 2023 and is in the process of receiving full approval from the FDA and making its way into the market. It exclusively binds to pyroglutamate Aβ—a key driver for plaque formation—to reduce Aβ build-up and can also delay cognitive decline in AD patients [65]. Although these antibodies represent a major shift in the disease-modifying treatment of early-stage AD patients, they have been associated with certain risks and challenges, such as amyloid-related imaging abnormalities (ARIA). However, ALZ-801 (Figure 3), which has tramiprosate as an active agent, showed anti-Aβ activity without binding to Aβ plaques and minimized ARIA risk. Early results from a phase-II trial have indicated its potential to delay memory decline in the early stages of AD, in patients on account of its possessing the APOE4 gene. As of May 2025, the phase-III clinical trial of ALZ-801 on the homozygous APOE 4 allele did not meet its desired goal across all the groups; however, a gradual slowdown of cognitive decline and brain atrophy was found in patients with mild cognitive impairment (MCI). This suggests that ALZ-801 might offer future treatment options, particularly to APOE4 homozygotes during the early stages of Alzheimer’s disease. Moreover, ALZ-801 could offer a safer and convenient alternative to the IV antibody, particularly for AD patients at high risk of ARIA, by inhibiting phosphorylated tau accumulation. Nevertheless, research is still ongoing to further analyze the data to reach a conclusion regarding the approval of ALZ-801 for MCI AD patients [66].

In addition, two antibodies that target the tau protein and inhibit its aggregation have entered clinical trials. Bepranemab (a recombinant, humanized, full-length immunoglobulin G4 monoclonal antibody) targets the central tau region and is currently undergoing phase-II trials, whereas E2814, a monoclonal antibody targeting the microtubule-binding region of tau protein, is currently undergoing phase-II and phase-III clinical trials, which are expected to conclude in 2027. Moreover, AL002 is another antibody currently under investigation in phase-II clinical trials that assists in clearing Aβ aggregation by increasing the microglial function [63].

A crucial aspect of combating neurodegenerative diseases extends beyond symptomatic treatment and concerns our capacity to influence neurogenesis by increasing the proliferation and differentiation of neural cells. This is associated with the activation of pathways such as MAPK ERK, PI3K/AKT, NFkB, Wnt, BDNF, and NPAS3. Low-molecular-weight compounds that affect proliferation/differentiation include LiCl, 4-aminothiazoles, pregnenolone, ACEA, harmine, D2AAK1, methyl 3,4-dihydroxybenzoate, and shikonin [67]. Given their anti-neurodegenerative properties, these compounds hold the potential to serve as lead compounds for the design of novel AD drugs.

**Table 2:** Therapeutic approaches for AD, including classical pharmacological interventions and emerging technologies. Each approach is characterized by its description, advantages, and disadvantages, providing insights into the diverse strategies employed to address the complex challenges associated with AD [68].

Approach Name	Description	Advantages	Disadvantages
Enzyme inhibitors	Targets enzymes like AChE to increase acetylcholine levels in the brain	Temporarily alleviates cognitive symptoms	Limited efficacy; does not modify disease progression
NMDA Receptor Antagonists	Antagonizes NMDA	Temporarily improves dementia symptoms	Side effects; limited long-term benefits
Gene Therapy	Modifies genes to address underlying causes of Alzheimer’s disease	Targets disease at a genetic level	Complex; potential ethical concerns
Lipid Nanoparticles	Utilizes nanoparticles for drug delivery, particularly for crossing the blood–brain barrier	Enhances drug delivery to the brain	Technical challenges; potential toxicity
Dendrimer-Based Therapy	Uses dendrimers for targeted drug delivery	Precise drug delivery; reduced side effects	Limited research; potential toxicity
Monoclonal Antibody Treatment Lecanemab Donanemab Aducanumab	Aims to clear Aβ fibrils/plaques	Disease-modifying effects Slows down cognitive decline in early AD	ARIA risk; high cost; controversial efficacy

### 2.3. Multi-Target Drug Design Strategy

The “one drug, one target” paradigm was unable to produce the intended therapeuic results for AD because of its complex pathomechanism. It may be possible to modulate the course of the disease, however, by employing multi-target-directed ligands (MTDLs), which involve a single multifunctional molecule that can affect many interrelated pathogenic pathways [69]. This method is considered more promising than traditional combination therapy because it preserves the diverse pharmacodynamic effects of multiple agents while offering additional advantages, such as a reduced risk of side effects, a simplified dosing regimen that enhances patient compliance, and a lower potential for drug-drug interactions. Thus, it has become widely recognized that multi-target drugs represent a potential treatment option for AD.

Promising example of MTDLs include dual AChE/MAO-B inhibitors, which have been shown to offer a number of benefits in AD by addressing imbalances in monoaminergic systems and cholinergic deficiencies simultaneously [70]. While dual compounds have shown neuroprotective potential in preclinical trials, their full potential for disease treatment remains to be established clinically [70]. A unique dual AChE/MAO-B inhibitor that has the potential to offer both symptomatic and disease-modifying effects, ladostigil, is being developed through phase-II clinical trials. This was achieved by Bhatia et al. using the MTDL drug design strategy, combining the pharmacophores of rasagiline, an MAO inhibitor, and rivastigmine into a single molecular entity [71]. Consequently, the idea of AChE/MAO-B as dual inhibitors has drawn a lot of attention lately, and many AChE and MAO-B dual inhibitors that have been created using the MTDLs technique [72]. Although AChE and MAO-B are well-established enzymes for their pathological roles in AD, recent findings underscore their potential as synergistic targets for therapeutic strategies. AChE is important for cholinergic neurotransmission, and AChE inhibitors aim to restore acetylcholine levels, whereas MAO-B contributes to the oxidative stress and MAO-B inhibitors focus on eliminating the stress and neuroinflammation [73]. Targeting both enzymes concurrently may offer synergistic effects addressing cognitive decline and neuroinflammation [74].

This review explores the functions and potential synergies that may result from simultaneous targeting AChE and MAO-B as it examines the relevance of both proteins in AD. In particular, this review provides an overview of the dual inhibitors of AChE and MAO-B, focusing on the design and structure–activity relationship and stressing major challenges in the field. The literature on dual inhibitors covers articles published from 2018 to 2024.

## 3. AChE and MAO-B as Selected Molecular Targets

### 3.1. Structural Aspects of AChE and MAO-B

**Figure 4:**
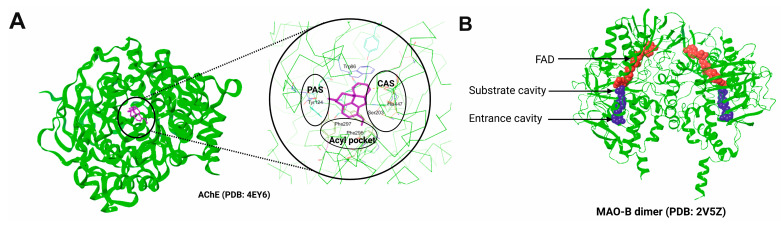
The crystallographic structures of AChE(PDB: 4EY6) (**A**) and MAO-B (PDB: 2V5Z) (**B**). Important sub-sites in the AChE active site are highlighted. The catalytic active site (CAS) is characterized by the presence of Ser203, His447, and Glu334; whereas the peripheral anionic site (PAS) helps in the entry and orientation of the ligand and majorly contains aromatic residues. Binding cavity of MAO-B is shown in surface representation.

The structural aspects of AChE and MAO-B are critical for understanding their role in AD pathology and designing drugs using this information. AChE is a cholinergic enzyme mainly found at the postsynaptic membranes, responsible for the hydrolysis of neurotransmitter acetylcholine into choline and acetate. Its structure features a deep narrow gorge of around 20 Å in length, composed of the active site along with multiple binding sub-sites that interact with distinct regions of the substrate (Figure 4A). The catalytic active site (CAS) is situated at the base of this narrow gorge known for its catalytic triad of three important residues, namely, Ser203, Glu334, and His447, to coordinate the process of acetylcholine hydrolysis. Contrary to the active site, the peripheral anionic site (PAS) is located at the entrance of the gorge and is critical to facilitate the movement of acetylcholine to the active site. Additionally, it might interact with other molecules, particularly Aβ, causing the aggregation of Aβ plaques [75]. Moreover, hydrophobic residues Trp86, Phe338, and Tyr337 contribute to substrate binding and stabilization within the anionic subsite [76]. The binding ability of the enzyme with respect to acetylcholine and related substrates is determined by these structural components [38]. ACh is hydrolyzed at the CAS site after being directed into it by the PAS via the cation-π interaction. Multiple refined structures of human AChE in complexes with inhibitors are available in the Protein Data Bank, such as 4EY6 [77], 5FPQ [78], 8DT7 [79], and 7E3D [80].

MAO-B is a flavoenzyme of 60 kDa consisting of two major domains: the FAD-binding domain and the substrate-binding domain. The former binds to flavin–adenine dinucleotide cofactor, which is critical for the oxidation of monoamine, whereas the substrate monoamine binds at the substrate-binding site. The substrate-binding site has an entrance cavity and a substrate cavity, where the former is large and hydrophobic in nature and the latter is relatively small and located adjacent to the FAD binding site (Figure 4B). Ile199 and Tyr326 residues split the binding pocket of MAO-B into the substrate cavity and entry cavity [74]. The binding site residues, which are crucial for substrate recognition and stabilization, are Tyr398 and Tyr435; however, Cys172 is known to maintain the structural integrity of the active site. In addition, Ile199 regulates the entry of ligands into the enzyme cavity. Moreover, a smaller hydrophobic entrance cavity next to the substrate cavity, lined with Tyr398 and Tyr435, could create an aromatic cage by engaging with the substrate’s amino group through π–cation interactions [72].

A few crystal structures of the MAO-B in complex with inhibitors such as 2V5Z [81] and 6FW0 [82] are accessible in the PDB repository. MAO-B catalyzes the deamination of neurotransmitters (in particular dopamine) by removing their amino group, with flavin adenine dinucleotide (FAD) serveing as a cofactor at a primary binding site. The binding pocket of MAO-B is divided into a substrate cavity and an entry cavity by the amino acid residues Ile199 and Tyr 326 [74]. To regulate the entry of ligands into the enzyme cavity, Ile199 residue for its flexibility is essential. Furthermore, a smaller hydrophobic entrance cavity next to the substrate cavity lined with certain residues, namely, Tyr398 and Tyr435, could create an aromatic cage by engaging with the substrates’ amino group through π-cation interactions [72].

π–π interactions between Trp86 and Phe295 were seen in the active site of AChE via aromatic rings. The phenyl ring of MAO-A/B produced π–π stacking interactions, with the aromatic cage formed by Tyr435 and Tyr398 in MAO-B, as indicated by the docking into the active site [74]. Both enzymes’ structural components are dynamic; i.e., they interact with water molecules. Water molecules are essential to the hydrolysis step in AChE, which speeds up substrate breakdown. Moreover, water molecules help to stabilize the FAD cofactor and facilitate substrate binding in MAO-B. The catalytic processes and general stability of AChE and MAO-B are further influenced by these interactions with water molecules [83].

### 3.2. Chemical Methods for Testing AChE and MAO Activity

#### 3.2.1. Ellman’s Method

The measurement of AChE activity can be based on a coupled reaction in which an acetylcholine analog substrate is hydrolyzed, and the resulting product participates in a secondary reaction that generates a measurable signal, such as a colorimetric, fluorometric, or chemiluminescent output. In chemiluminescent assays, light emission is produced during a chemical reaction and detected by a luminometer. In colorimetric assays, changes in absorbance at specific wavelengths are used to quantify AChE activity.

**Scheme 1:**
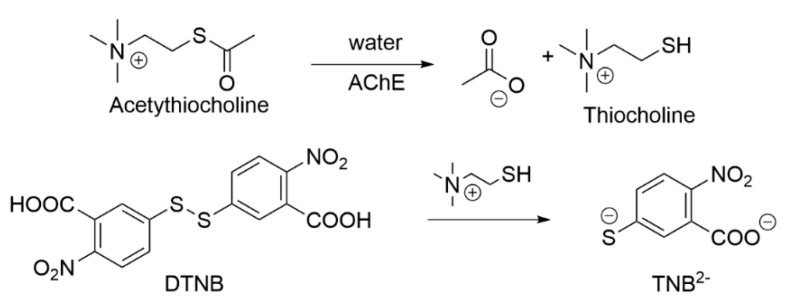
Ellman’s method.

The most commonly used method is Ellman’s method (Scheme 1) [84]. This involves the deacylation of acetylthiocholine in the presence of AChE, resulting in the in situ formation of a thiol that reacts with Ellman’s reagent, 5,5′-dithiobis-(2-nitrobenzoic acid) [DTNB]. The resulting TNB^2−^ ion is quantified using a spectrophotometer by measuring absorbance at a wavelength of 412 nm, applying an absorption coefficient dependent on the dilution factor of the buffer solutions. The change in visible light absorbance is linearly related to the change in enzyme activity. By modifying the Ellman’s method, butyrylcholinesterase activity can also be measured [85]. In this case, butyrylthiocholine is used as a substrate, which hydrolyzes catalytically to thiocholine and butyric acid. Catalytic concentration is determined based on the rate of the decrease in hexacyanoferrate (III) at a wavelength of 405 nm. Other methods operate on a similar principle.

#### 3.2.2. Other Methods for Testing AChE Activity

**Figure 5:**
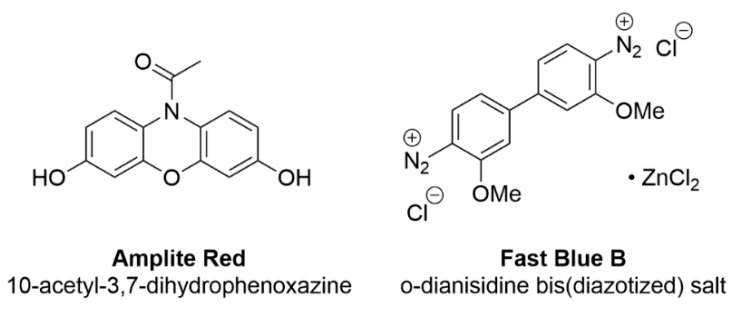
Colored indicator substances.

Alternatives to Ellman’s method involve changing the dye reagent and modifying the second reaction step. The choline produced in the catalytic hydrolysis reaction can be oxidized by choline oxidase to betaine aldehyde and hydrogen peroxide. In the next step, peroxidase converts hydrogen peroxide and Amplite Red/Amplex Red (Figure 5) into resorufin (excitation at 540 nm; emission at 590 nm). Similarly, the produced thiocholine reacts with a proprietary probe, Thiolite Green, to produce a fluorescent adduct (excitation at 490 nm; emission at 520 nm) [86].

**Scheme 2:**
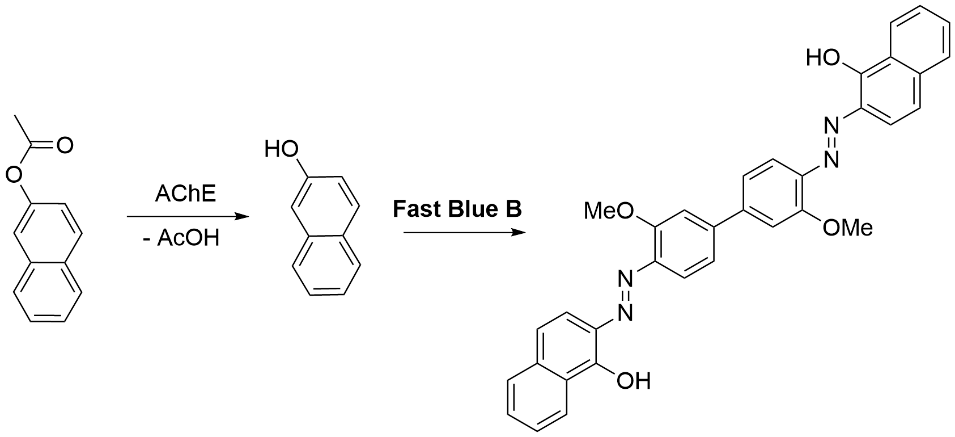
AChE activity test using Fast Blue B [87].

Another noteworthy method involves using β-naphthyl acetate as a substrate in the catalytic reaction, with Fast Blue B as the dye reagent, resulting in a stable purple color (Scheme 2) [87].

**Scheme 3:**
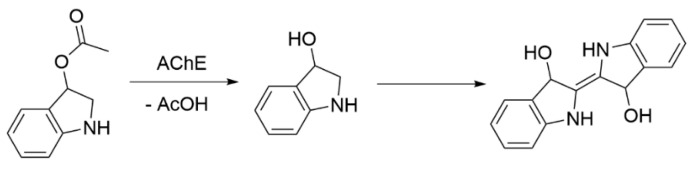
AChE activity test using indoxyl acetate as a substrate [88].

Using indoxyl acetate in ethanol as the substrate allows for measuring the absorbance of the mixture at a wavelength of 670 nm (Scheme 3) [88].

Among other methods for determining AChE activity, the electrometric method by Frawley and the colorimetric method by Pilz can be mentioned. Cholinesterase activity can also be determined by the Hestrin method by detecting the acidic complex of Fe^3+^ ions with acetylhydroxamic (or benzoyl) acid, which are formed by the unhydrolyzed portion of acetylcholine after reacting with hydroxylamine in an alkaline environment [89,90,91].

#### 3.2.3. MAO-B Inhibition Assays

The study of MAO-B enzymatic activity is based on principles similar to those used in AChE analysis. In this case, the most commonly used method is also fluorescence-based, where the amount of colored product, quantitatively linked to the hydrogen peroxide produced in the enzymatic reaction, is measured.

**Scheme 4:**
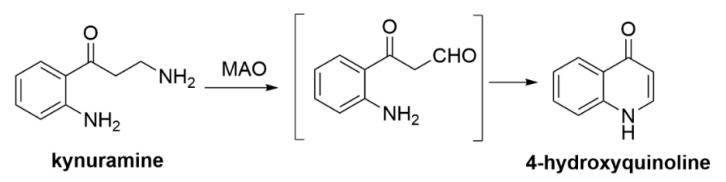
MAO-B inhibition assays with kynuramine [92].

As early as the 1960s, a method utilizing kynuramine as a substrate was proposed. The oxidation reaction first produces an intermediate product—an aldehyde, which then undergoes intramolecular cyclization to form 4-hydroxyquinoline (Scheme 4) [92]. Among the historical methods for determining MAO-B activity, the use of L-[^11^C]-deprenyl with positron emission tomography (PET) is also noteworthy [93,94].

**Scheme 5:**
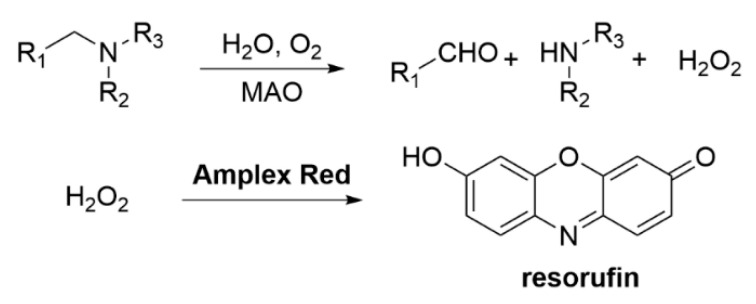
MAO-B inhibition assays with Amplex Red.

Analogously to the study of AChE activity, Amplite Red can be used to study MAO-B. The action of the oxidase releases hydrogen peroxide, which is then used to convert Amplex Red to resorufin (Scheme 5) [95].

## 4. Overview of Dual AChE and MAO-B Modulators

**Figure 6:**
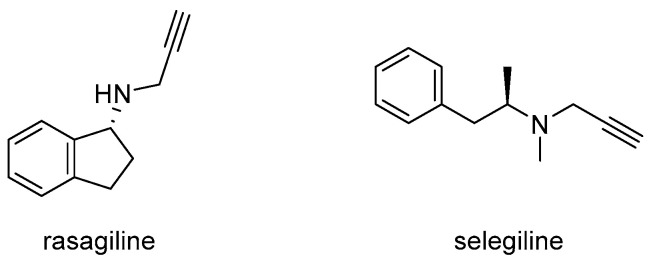
Rasagiline and selegiline.

A variety of methods, including fusion, linkage, and the hybrid approach, have been used in the search for potent dual inhibitors for AD therapeutics. One major area of research is improving cholinergic neurotransmission, which includes developing AChE inhibitors such as donepezil and rivastigmine to increase acetylcholine levels and slow down cognitive aging [96]. In addition, compounds like rasagiline and selegiline (Figure 6) are being studied as potential targets for MAO-B, offering an approach to controlling neurotransmitter levels and alleviating the oxidative stress associated with AD [97].

In recent years, the strategy of designing dual MAO-B/AChE inhibitors has become profoundly important in the treatment of AD. Among the new inhibitors are the following:

- Modified structures of known active substances (rasagiline, rivastigmine, selegiline, donepezil, tacrine);
- Carbamate derivatives of *N*-propargylaminoindans and *N*-propargylphenethylamines;
- Chalcone-based inhibitors;
- Coumarin-based inhibitors;
- Chromone-based inhibitors;
- Benzo-5-membered ring-based inhibitors;
- Imine and hydrazone inhibitors;
- 3,4-dihydropyrimidin-2(*1H*)-one inhibitors;
- Pyridoxine-based inhibitors;
- 1-(*2H*)-phthalazinone-based inhibitors;
- Diclofenac derivatives.

### 4.1. Chalcone Derivatives

**Figure 7:**
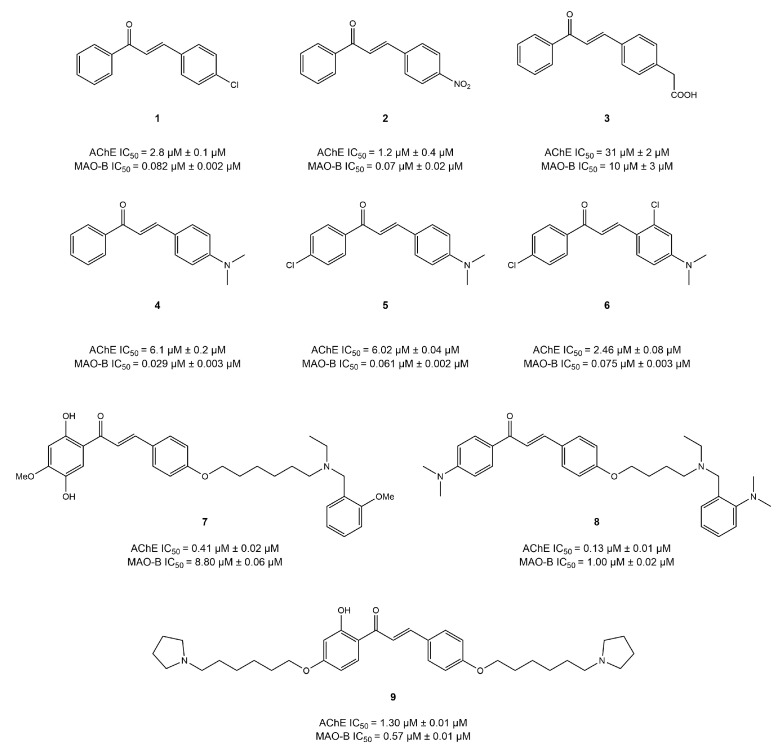
Chalcone derivatives [101,102].

Chalcone is an open-chain flavonoid commonly found in several plants, consisting of two benzene rings linked with three carbon atoms. Various structural modifications of the chalcone core structure have been explored to develop chalcone analogues that can target MAO-B and AChE in the treatment of AD [98]. Oh et al. [99] tested six natural and six synthetic chalcones for their dual inhibitory potential against AChE and MAO-B using the fusion technique. Their study reported that five synthetic chalcones (Figure 7) moderately inhibited AChE, with IC_50_ values ranging from 1.2 to 6.07 μM. However, these compounds showed strong inhibition of MAO-B, exhibiting IC_50_ values ranging from 0.029 to 0.082 μM. Molecular docking results revealed that all five compounds showed strong binding affinities with hMAO-B compared to the AChE. Additionally, the carbonyl oxygen of these five chalcones displayed hydrogen bonding with Cys172 of hMAO-B, whereas no hydrogen bonds were seen in the AChE pocket. Moreover, one synthetic chalcone **3** showed poor activity against both the enzymes, and it also lacked the hydrogen bonding with Cys172 of hMAO-B, probably due to the charge effect by the carboxymethyl group in the structure. In addition, docking simulations revealed that different R groups at C-4 position, such as methyl, dimethylamine, and methoxy, increased the selectivity of compounds for MAO-B. Moreover, the cyclization of ring A with the benzothiazine ring causes a sharp decline in the inhibitory potential of both enzymes. However, the cyclization of ring A using 1,3-dioxolane and the replacement of ring B with a thiophene ring potently increased the activity value against AChE [100]. The study in question suggested compound **2** as a promising lead compound, showing balanced dual inhibition for both enzymes [72,99].

**Figure 8:**
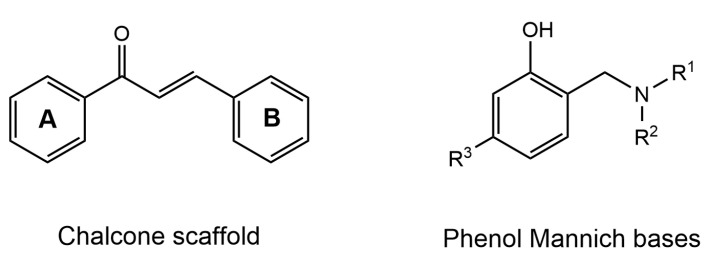
Structure of chalcone scaffold and phenol Mannich bases.

Sang et al. [101] synthesized several chalcone and donepezil-based compounds to evaluate their dual-balanced activity against AChE and MAO-B. In their study, several compounds were presented (Figure 8). Compound **7** exhibited decent AChE-inhibitory activity (IC_50_ = 0.41 μM) and moderate inhibition of MAO-B (IC_50_ = 8.8 μM). The molecular docking results revealed that compound **7** was positioned near the CAS and PAS of AChE, where Ser122, Tyr334, and Arg289 were observed to be actively involved in hydrogen bond formation. In addition, Phe330, Phe288, Arg289, Tyr334, and Gly335 were noticed to form hydrophobic interactions. Moreover, compound **7** showed fairly stable interactions with the key binding residues of MAO-B, such as Leu345 and Ala325. Additionally, Ala325, Thr201, and Tyr326 were observed to make H-bond and Tyr326, and Tyr60, Leu345, and Glu84 were involved in hydrophobic interactions [101]. In anticipation to design improved dual inhibitors for the treatment of AD, compounds **8** and **9** were synthesized by the same group via a linkage approach. Both compounds exhibited antioxidant potential in addition to their improved and balanced activities. Compound **8** [102] showed an IC_50_ = 0.13 and 1.0 μM, whereas compound **9** [103] exhibited IC_50_ = 1.3 and 0.57 μM for AChE and MAO-B, respectively. Molecular docking analysis showed the compound **8** could fit into the CAS of AChE and establish π–π interaction with Trp286 and Tyr341. In addition, Tyr314 was observed to form a hydrogen bond with the hydroxyl group of A ring. Moreover, hydrophobic interactions were also observed with the Tyr337, Trp86, and Trp286 of AChE binding pocket. Additionally, a 2-hydroxy-acetophenone moiety of compound **8** established two hydrogen bonds with the Cys172 and Phe168 of MAO-B, while ring A and B generated hydrophobic interactions with Tyr435 and Leu171 [102]. Likewise, compound **9** was docked into both targets, and unlike compound **8**, it fully occupied the pocket of AChE. A hydrogen bond was observed between the oxygen atom of ring B and Phe288, whereas ring A formed a π–π interaction with Phe330. In addition, it generated hydrogen bonding with the Cys172, Ile199, Ile198, and Gln206 of MAO-B, while Tyr435 and Tyr398 formed a π–π interaction [103].

Phenolic Mannich bases (Figure 8) have been modified with respect to the chalcone core structure using the fusion approach in several research groups because of their potential neuroprotective and antioxidant effect.

**Figure 9:**
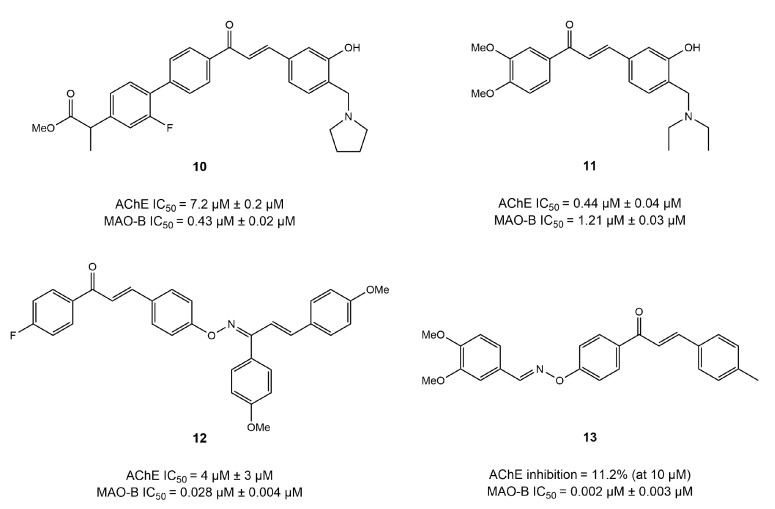
Mannich bases [104,105,106].

Tian et al. reported that compound **10** exhibited IC_50_ = 7.15 and 0.43μM for AChE and MAO-B, respectively [104]. The docking of this compound into AChE (PDB:1EVE) revealed that Ser286 formed a hydrogen bond with the phenylacetate group, whereas piperidine established a hydrogen bond through Asn85 of CAS and a salt bridge with Asp72. Moreover, the docking results with respect to the MAO-B (PDB:2V5Z) showed that Gly101 was involved in generating a hydrogen bond with the B ring of compound **10**, and Glu84 formed a salt bridge with the piperidine fragment. However, the A ring bound to the Ile199 of the entrance cavity and phenyl group formed π–π interactions with Tyr398 in the substrate cavity [104]. In a similar study [105], another chalcone–Mannich compound **11** showed IC_50_ values of 0.44 and 1.21 μM for AChE and MAO-B, respectively. It was observed to bind with both of the binding sites of CAS and PAS of AChE in molecular simulation analysis. Moreover, in the pocket of MAO-B, Tyr435 residue established a hydrogen bond with the carbonyl moiety, and Ile199 formed a hydrogen bond interaction with a hydroxyl group of the B ring, whereas Tyr398 generated π–π interactions with the A ring of compound **11** (Figure 9) [105].

The inhibitory effects of chalcone oxime ethers on AChE and MAO-B have been investigated in various studies for their potential as AD drugs. In an interesting study, Oh et al. [106] utilized a fusion approach to synthesize a series of chalcone-based oxime ethers and explored their activity on both AChE and MAO-B enzymes. The results reported that compound **12** displayed the most balanced inhibition potential of IC_50_ = 4.39 and 0.028 μM against AChE and MAO-B, respectively [106]. A SAR study of dimethoxy chalcone revealed that a substitution in the acetophenone moiety of chalcone, instead of (-H) or (-OMe), caused excellent inhibition of MAO-B. It can be suggested from these results that electronegative groups in the acetophenone portion of chalcone significantly increase the MAO-B inhibition potential. Additionally, SAR analysis discovered that modification in the A ring of chalcone with a dioxime ether (compound **13**) led to a slight increase in MAO-B inhibition (IC_50_ = 0.018 μM) but a significant decline in the inhibitory potential of AChE (IC_50_ = 10 μM). The molecular docking results further supported their findings, where compound **13** displayed the highest binding affinity in the binding pocket of MAO-B.

**Scheme 6:**
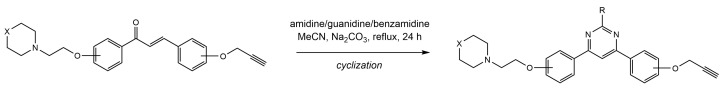
Cyclization of the chalcone scaffold [107].

**Figure 10:**
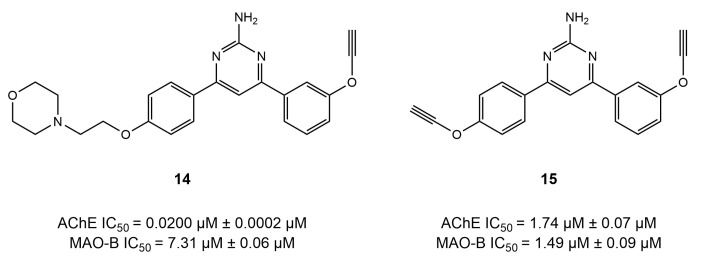
Impact of the chalcone cyclization on AChE- and MAO-B-inhibitory activities [72].

In a nutshell, the SAR study of chalcone-based derivatives revealed that substitutions of the A ring at position 2 (hydroxyl and alkoxyl) and position 4 (morpholine, piperazine, oxime ether, and cyclization into 10-H phenothiazine) result in a decrease in AChE inhibition potential. However, morpholine and piperazine decrease MAO-B activity, while oxime ethers increase the inhibitory potential of MAO-B. Additionally, substitutions of the B ring at position 3 (oxime and carbamate) and at position 4 with -OH and carboxymethyl decrease the activity of the AChE enzyme. In contrast, the chlorine, nitro groups, and aminoalkyl groups caused an increase in the MAO-B-inhibitory potential at position 4 of the B ring of the chalcone scaffold. Moreover, the A ring was observed to interact with the Ser122, Tyr314, Trp286, Arg289, and Phe330 of AChE and the Tyr398 and Tyr435 of MAO-B. In addition, the Trp286 and Phe288 of AChE and the Cys172, Ile199, and Tyr326 of MAO-B facilitated the binding of the B ring of the chalcone moiety by establishing hydrogen bonding and π–π interactions [72]. It is also worth mentioning that the three-carbon fragment of the chalcone can be cyclized (Scheme 6), resulting in compounds **14** and **15**, with potent selective AChE inhibition but weak activity against MAO-B. The morpholine fragment appears to be essential for strong AChE inhibition (Figure 10) [72].

### 4.2. Coumarin Derivatives

Coumarins, heterocycles with a 1,2-benzopyranone scaffold commonly found in several plants, have been found to exhibit anti-AChE and MAO-B activities [108]. Several research studies have reported the potential of natural and synthesized coumarin derivatives in targeting and inhibiting both AChE and MAO-B [72].

**Figure 11:**
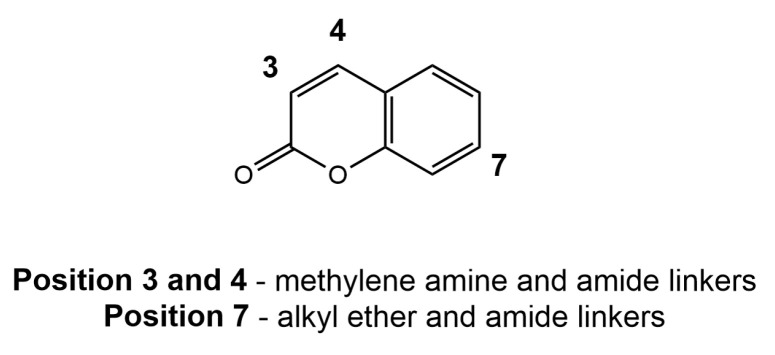
The site of the attachment of substituents in the modification of coumarins.

A study by Vina et al. [109] explored the modifications on the phenyl ring in the 2-position of coumarins with an arylamide or an alkyamide linker by utilizing the linkage approach. Substitution with benzylamine derivatives at the 3- or 4-position of the coumarin scaffold significantly enhances AChE-inhibitory activities. However, replacing benzylamine groups at the 3-position with benzamide moieties sharply decreases AChE-inhibitory activities, while remarkably increasing MAO-B-inhibitory activities. The introduction of the α,β-unsaturated ketone moiety at the 3-position reduces AChE-inhibitory activities. Substitution with benzamide moieties at the 7-position hinders the development of balanced AChE and MAO-B dual inhibitors, whereas introducing flexible AChE-inhibitory pharmacophores at the same 7-position is advantageous for obtaining potent and balanced AChE and MAO-B dual inhibitors. Moreover, *N*-benzylpiperidine or *N*-alkyloxy moieties at the 7-position have no significant effect on AChE inhibition but exhibit a remarkable impact on MAO-B inhibition, suggesting a greater influence of the protonatable portion decoration on MAO-B affinity compared to AChE (Figure 11).

**Figure 12:**
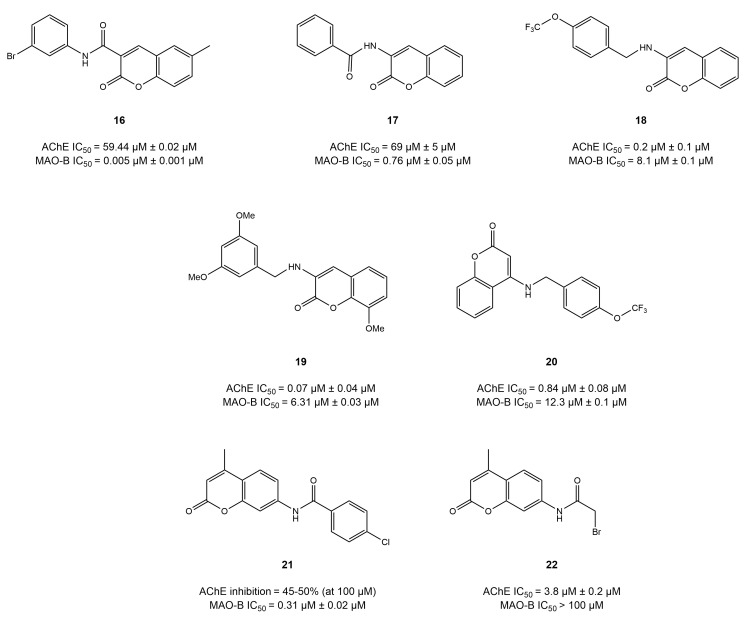
Coumarins derivatives [109,110,111].

The study results reported that this modification (compound **17**) resulted in poor AChE inhibition (IC_50_ = 69.47 μM) but potent MAO-B inhibition (IC_50_ = 760 nM) [109]. Interestingly, similar results were reported by another study for the modifications at position 3, where amide linkers (compound **16**) significantly decreased AChE inhibition potential (IC_50_ = 59 μM) and remarkably increased MAO-B inhibition (IC_50_ = 4.6 nM) [72]. However, the results from Zhang et al. [110] indicated a balanced inhibition potential of two modified coumarins with an amide linker at position 3, signifying the importance of alkylamine linkers for the development of coumarin-based dual inhibitors. Compound **18** displayed IC_50_ values of 0.163 and 8.13 μM for AChE and MAO-B, respectively, whereas compound **19** exhibited IC_50_ values of 0.068 and 6.31 μM against AChE and MAO-B, respectively. Moreover, modification at the 4-position of the phenyl ring (compound **20**) resulted in remarkable AChE-inhibitory potential (IC_50_ = 0.84 μM) but poor inhibition of MAO-B (IC_50_ = 12.31 μM). Rodríguez-Enríquez et al. [111] explored the amide modifications at the 7-position of the coumarins scaffold and evaluated their dual inhibition potential against AChE and MAO-B. Modification with an aromatic amide in compound **21** at position 7 showed no significant activity against AChE. Surprisingly, the substitution of the fatty acid amide at the 7-position of compound **22** removed the inhibition potential of MAO-B (IC_50_ > 100 μM) (Figure 12).

**Figure 13:**
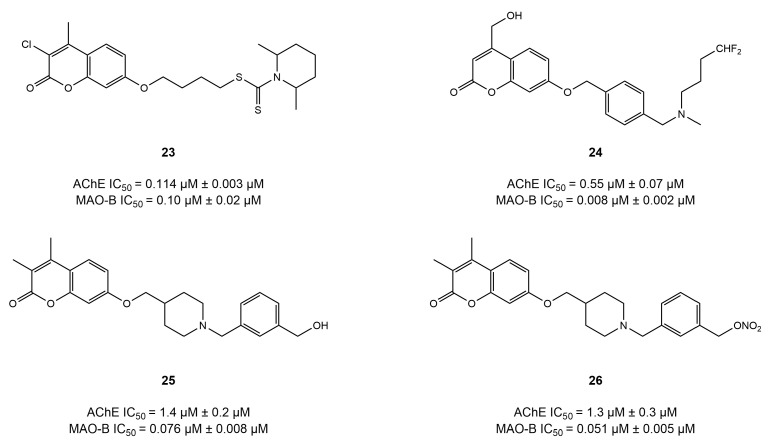
Coumarin-based ethers [112,113,114].

A series of coumarin-based dithiocarbamates was synthesized and evaluated by He et al. [112] for their potential to target AChE and MAO-B in the treatment of AD (Figure 13). Among all the compounds tested, compound **23** showed a balanced activity of IC_50_ = 0.114 and 0.101 µM for AChE and MAO-B, respectively, suggesting the significance of modifications at position 7 in coumarins. Likewise, compound **24** [113] and compound **25** [114] (as well as a similar derivative **26**) displayed IC_50_ values of 0.55 and 1.4 μM for AChE and IC_50_ = 0.008 and 0.076 μM for MAO-B. The contrasting results among these studies and the study conducted by Rodríguez-Enríquez et al. could be explained by the ability of former compounds to reach and interact with the binding pocket of AChE (CAS) and MAO-B. Docking simulations highlighted that the coumarin core interacted with the key residues of AChE (Trp86, Gly121, Trp286, Tyr341, Tyr337, Gly447, and Gly448) and MAO-B (Tyr60, Pro102, Pro104, Phe343, Tyr398, and Tyr435).

Overall, SAR analysis revealed that substitution with benzylamine groups at the 3- or 4-positios of the coumarins’ core remarkably increases AChE inhibition potential. However, modifications at position 7, including N-benzylpiperidine or N-alkyloxy, enhances the balanced inhibition protentional of the both the AChE and MAO-B receptors.

### 4.3. Chromone Derivatives

**Figure 14:**
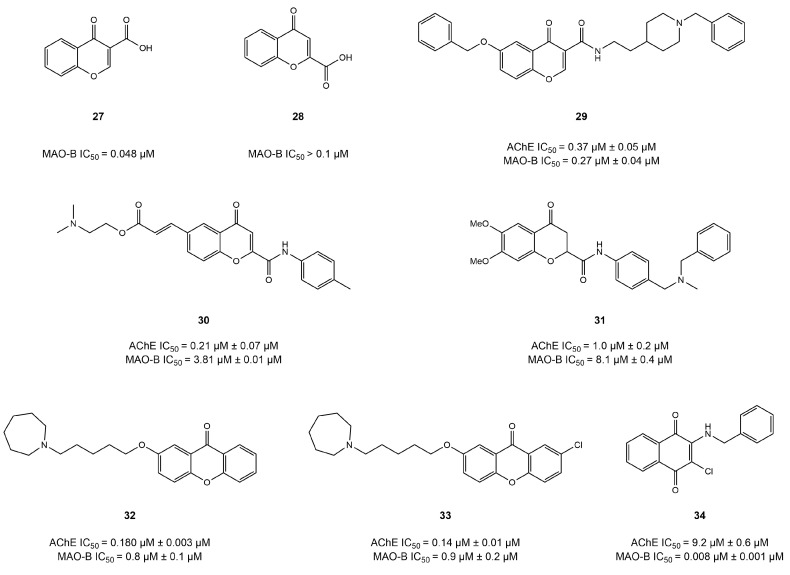
Chromone derivatives [115,116,117,118,119,120].

An isomer of coumarin called chromone provides an important scaffold in the field of dual AChE/MAO-B inhibitors. All the chromone derivatives described below are shown in Figure 14.

Positional modifications in the core structure of chromone with carboxyl groups have been investigated by Carradori et al. [115]. The results indicated that the introduction of the carboxyl group at position 3 of the chromone scaffold (compound **27**) greatly contributes towards selective inhibition of MAO-B (IC_50_ = 48 nM). This outstanding activity might be explained via the hydrogen bond formed between Tyr326 of MAO-B and the carboxyl group of compound **27**. However, a significant decline in activity (IC_50_ > 100 nM) was observed when carboxyl group was substituted at position 2 of the chromone core in compound **28**.

In addition to their MAO-B inhibition, research has focused on obtaining multi-target chromone derivatives that could exhibit balanced AChE-inhibitory potential for the treatment of AD. In an interesting study, Wang et al. [116] synthesized hybrid chromone derivatives by using a fusion approach to introduce the AChE-inhibitory pharmacophore into the chromone structure. Among the compounds tested, compound **29** showed the highest inhibitory potential for AChE and MAO-B. IC_50_ = 0.37 and 0.27 μM, respectively, suggesting that *N*-benzylpiperidine substitution might be significant to the development of chromone-based dual inhibitors. Docking simulations showed the *N*-benzylpiperidine of compound **29** established interactions with the Trp84 and Phe330 of AChE and occupied the entrance cavity of MAO-B. Additionally, the amide carbonyl group formed a hydrogen bond with Tyr326, and the benzyloxy group was involved in forming π–π interactions with the Tyr398 and Tyr435 of MAO-B.

Estrada-Valencia et al. substituted carboxamide at position 2 of the chromone scaffold and obtained compound **31**, which showed balanced inhibitory potential for AChE and MAO-B (IC_50_ = 1.0 and 8.1 μM) [117]; however, the carboxyamide substitution at the same position of the chromone core in compound **30** resulted in strong AChE activity (IC_50_ = 0.21 μM) but comparatively poor MAO-B inhibition potential (IC_50_ =3.81 μM) in a similar study [118]. This suggests that modification at position 2 of the chromone core, particularly with carboxamide, might lead to potent AChE inhibition potential.

Łażewska et al. [119] cyclized the chromone structure using the incorporation approach and developed a series of dual chromone-based inhibitors of AChE and MAO-B. They reported two compounds (**32** and **33**) with an IC_50_ = 180 and 136 nM for AChE and IC_50_ = 775 and 897 nM for MAO-B, respectively. A docking study with AChE showed that the alkyl group of the ligand formed hydrophobic interactions with Phe290 and Phe330, whereas the azepane ring was observed to generate interactions with Trp84, Phe330, and His440. In addition, the xanthone moiety occupied the PAS of the AChE pocket and formed hydrophobic interactions with Tyr70, Tyr121, and Trp279.

In another study, Campora et al. [120] evaluated two series of chromone-based anthraquinones and naphthoquinones modified with aromatic chains as potential therapeutics for AD. Compound **34** displayed balanced AChE and MAO-B inhibition potential (IC_50_ = 9.2 and 0.0077 μM), respectively. Compound **34** was developed by replacing the oxygen atom with a carbonyl group in the pyranone ring of the chromone core. Molecular docking was employed to obtain insight into the binding interaction pattern, which revealed that the carbonyl group established hydrogen bonding with Tyr72 and Tyr124 in the AChE pocket. Moreover, the carbonyl group was engaged in hydrogen bonding in the binding pocket of MAO-B through Gln206, Tyr326, and Tyr435.

**Figure 15:**
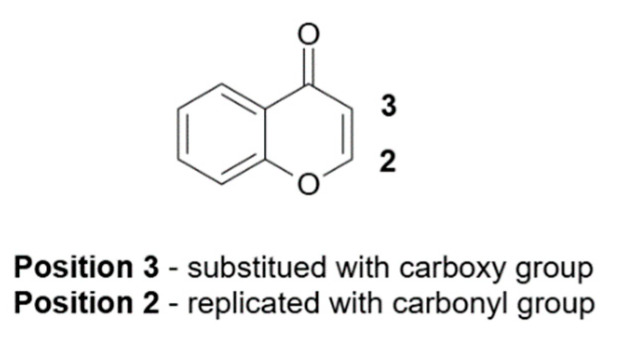
Structural modifications of chromone-based inhibitors.

Overall, the SAR studies of several substitutions at different positions of chromone-based series greatly impact the inhibition potential of both enzymes (AChE and MAO-B). The introduction of AChE-inhibitory pharmacophores onto the chromone scaffold, with a formamide group as a linker at the 3-position, leads to the creation of balanced dual inhibitors of AChE and MAO-B. Substitution with a formamide moiety at the 2-position of the chromone core, alongside the introduction of AChE-inhibitory pharmacophores at the 6-position, increases AChE-inhibitory activities but decreases MAO-B-inhibitory potencies. Donepezil-like pharmacophores at the 2-position, using a formamide moiety as a linker, result in a decrease in both AChE- and MAO-B-inhibitory activities. Conversely, introducing donepezil-like pharmacophores at the 3-position increases both AChE- and MAO-B-inhibitory activities (Figure 15).

The easily modifiable chromone nucleus allows for the creation of novel scaffolds (xanthone and naphthoquinone) through cyclization or core-hopping strategies. The introduction of a flexible alkoxy amine at the 2-position of xanthone increases AChE- and MAO-B-inhibitory activities, while the benzylamino group at the 2-position of naphthoquinone significantly enhances MAO-B-inhibitory activity [72].

### 4.4. Imine and Hydrazone Derivatives

**Figure 16:**
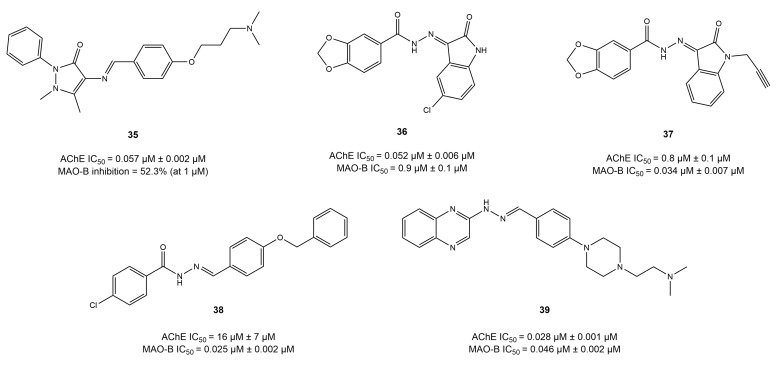
Imine and hydrazone derivatives [121,122,123,124,125].

It is well known that the C=N group, especially when it comes to MAO-B inhibition, is a powerful regulator of how inhibitors and enzymes interact. Derivatives of imine and hydrazone with the C=N group have shown promise as MAO-B inhibitors as the imine nitrogen forms important hydrogen bonds with Tyr407 [121]. Moreover, the potential of C=N as a potent linker to develop dual AChE and MAO-B have been widely studied (Figure 16).

By employing the linkage technique, several studies have developed imine-based anti-AD compounds that showed unbalanced dual-target inhibitory potency exhibiting strong MAO-B inhibition but little AChE inhibition [72]. However, compound **35** exhibited strong AChE inhibition (IC_50_ = 0.057 μM) but poor MAO-B activity, i.e., 52% at 1 mM [122].

Moreover, a handful of studies have reported on a few compounds (**36, 37**) with potent dual inhibition when hydrazone was used as a linker through the linkage approach [121,123]. The hydrazones derived from piperonylic acid presented balanced dual inhibition, compounds **36** and **37**, with IC_50_ = 0.052 and 0.85 μM for AChE and IC_50_ = 0.89 and 0.034 μM for MAO-B, respectively. However, compound **37**, bearing propargyl substitution, displayed the highest MAO-B inhibition (IC_50_ = 0.034 μM), suggesting the importance of propargyl substitution in enhancing MAO-B activity. Moreover, docking studies revealed that compound **37** established four hydrogen bonds with Gly121, Gly122, Ala204, and Phe295 and Ile198, Ile199, Gln206, and Tyr326 in the pocket of AChE and MAO-B, respectively.

Palakkathondi et al. synthesized fourteen arylhydrazide derivatives using the linkage approach and investigated their dual inhibition potential against AChE and MAO-B [124]. Among all the studies considered, compound **38** exhibited remarkable MAO-B activity (IC_50_ = 0.025 μM) but weak AChE potential (IC_50_ = 16.5 μM). The weak inhibition of AChE could be explained by the absence of AChE pharmacophore. Taking this into account, Xu et al. [125] introduced the AChE-inhibitory pharmacophore by using the linkage approach to obtain quinoxaline-based hydrazone dual inhibitors. Compound **39** displayed balanced potent dual inhibition, with IC_50_ = 0.028 and 0.046 μM for AChE and MAO-B, respectively. Docking analysis highlighted that hydrazone formed two hydrogen bonds with Phe295 and Arg296, whereas quinoxaline interacted with the Trp286 of AChE. However, in the pocket of MAO-B, quinoxaline formed π–π interactions with Tyr435, and hydrazine established a hydrogen bond with Glu206.

### 4.5. Benzo Five-Membered Ring-Based Dual Inhibitors

Heteroaromatic organic compounds such as indole and benzofuran serve as a core structure in many lead compounds and are widely studied structural units in pharmaceuticals [72]. In this section, we summarize the dual inhibitors that contain extended heterocyclic aromatic fragments in their structure, including indole, benzothiazole, benzimidazole, isobenzofuran, and thiazolopyrimidine moieties.

**Figure 17:**
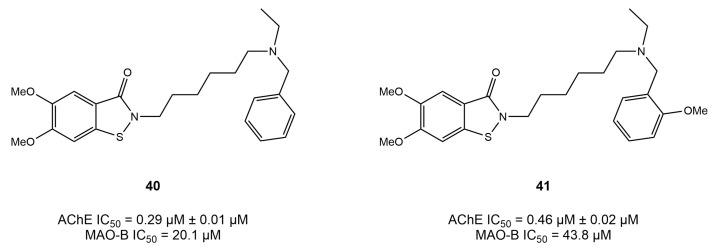
Isothiazolidinone derivatives [126].

Xu et al. [126] synthesized a series of compounds based on benzo[d]isothiazol-3(*2H*)-one- using a linkage approach and investigated their potency as multi-target agents for the treatment of AD. The results showed that most of the compounds displayed potent and selective activity for AChE. However, two compounds, **40** and **41** (Figure 17), displayed balanced dual inhibition, with IC_50_ values of 0.29 and 0.46 μM for AChE and IC_50_ values of 20.1 and 43.8 μM for MAO-B.

These derivatives possessed high AChE activity but low MAO-B inhibition, which could be attributed to their ability to fully occupy the AChE pocket, as suggested by docking simulations. Moreover, benzo[d]isothiazol-3(2*H*)-one- was observed to interact with Phe288 and Phe331 via π–π interactions, whereby a hydrogen bond was established by the Tyr121 of PAS with the *S* atom.

In a recent study, Liu et al. [127] synthesized a series of Mannich bases based on isobenzofuran-1(3*H*)-one and reported that compound **42** displayed a relatively balanced dual inhibition, with IC_50_ values of 0.041 and 0.30 μM for AChE and MAO-B, respectively. In a molecular docking study of AChE, the phthalide moiety formed π–π interactions with Tyr334, whereas several other residues, including Trp84 and Phe330, were observed to interact with compound **42.** Moreover, the benzyl group occupied the entrance cavity of MAO-B and interacted with prominent amino acid residues such as Cys172 and Il199.

Similarly, recently, a series of thiazolopyrimidine-based derivatives were synthesized using a linkage approach and characterized for their dual activity on AChE and MAO-B in AD therapeutics [128]. As a result, compound **43**, an indole-based derivative, has been reported to exhibit balanced dual inhibitory potential of IC_50_ = 0.042 and 0.33 μM for AChE and MAO-B, respectively. Moreover, Denya et al. developed a series of indole-based derivatives was by fusing the carbamate moiety with the indole core by using the fusion approach to investigate their multi-target potential [129]. They reported two compounds, **44** and **45**, which presented balanced and potent inhibition of AChE (IC_50_ = 3.70 and 2.41 μM) and MAO-B (IC_50_ = 2.62 and 1.84), respectively. Docking insights showed carbamate to be involved in forming a hydrogen bond with Trp84 in the CAS of AChE, whereas the indole group generated π–π interactions with Phe330 and Trp334. However, both derivatives occupied the substrate cavity of MAO-B and interacted with FAD through hydrogen bonding and π–π interactions.

**Figure 18:**
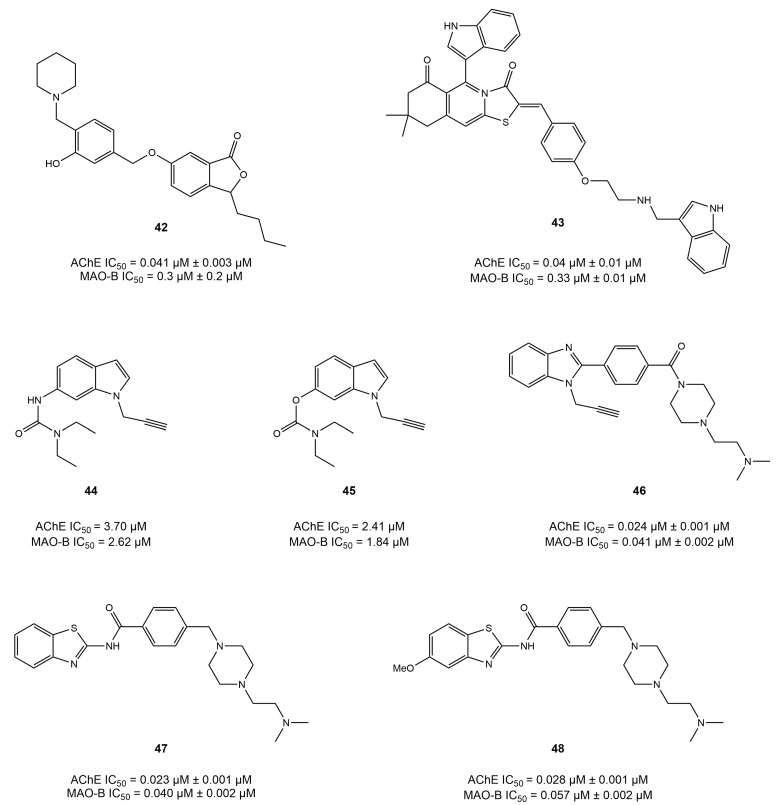
Aromatic heterocyclic-based derivatives [84,128,129,130,131].

In a similar study, Osmaniye et al. [83] developed a series of benzimidazole derivatives using the fusion approach to investigate potential dual inhibitors of AChE and MAO-B in AD treatment. They reported that compound **46** displayed extraordinary balanced activity against both enzymes (AChE IC_50_ = 0.024 μM; MAO-B IC_50_ = 0.041 μM). Moreover, the compound showed interestingly similar activity to that of donepezil and selegiline, as reported by in vitro methods. The molecular docking of compound **46** in both structures revealed that the benzimidazole ring generated π–π interactions with the Trp286 of AChE and Trp119 of MAO-B, respectively. In addition, the carbonyl group established a hydrogen bond with Tyr 345 in the binding pocket of MAO-B. Moreover, more recently, two compounds, **47** and **48** [130], were reported in a fusion study to synthesize benzothiazole-based multi-target derivatives for AD therapeutics. Compound **47** displayed IC_50_ values of 23.4 and 40.3 nM for AChE and MAO-B, respectively, whereas compound **48** showed IC_50_ values of 27.8 and 56.7 nM for AChE and MAO-B, respectively. The benzothiazole ring was observed to form π–π interactions with Trp286 and a hydrogen bond with Pro102 of AChE and MAO-B, respectively. All the dual inhibitors mentioned in this chapter are presented in Figure 18.

### 4.6. Diverse Scaffold-Based Dual Inhibitors

**Figure 19:**
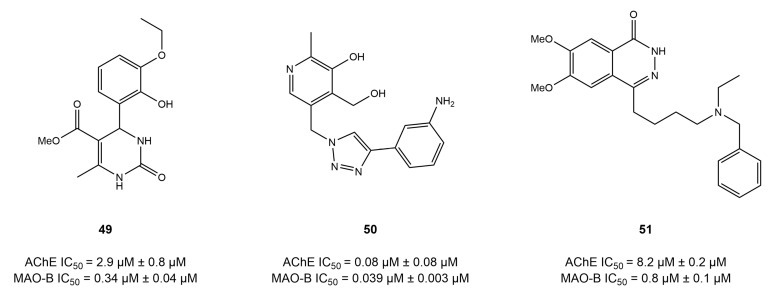
Diverse scaffold-based dual inhibitors [131,132,133,134].

In addition to the inhibitors described, several other diverse scaffold structures have been investigated in different studies as possible treatments of AD (Figure 19).

The 3,4-dihydropyrimidin-2(*1H*)-one-based dual compound **49** [135] demonstrated strong MAO-B inhibition (IC_50_ = 0.34 μM) and an average AChE inhibition potential (IC_50_ = 2.86 μM). Likewise, a group of researchers [132] utilized a linkage approach to synthesize the pyridoxine scaffold-based compound **50,** which exhibited strong and balanced inhibitory effects against AChE (IC_50_ = 0.081 μM) and MAO-B (IC_50_ = 0.039 μM). Molecular modeling analysis of compound **50** revealed that it generated π–π interactions with the Tyr398 and Tyr435 of AChE; however, the Gln206, Ile199, and Tyr69 of MAO-B were observed to form interactions with compound **50**.

Youdim [133] designed inhibitors that exhibited strong AChE-inhibitory action and a special ability to release the active molecule for MAO-B inhibition and metal chelation. Compound **51**, based on the 1(*2H*)-phthalazinone scaffold, showed strong MAO-B-inhibitory action (IC_50_ = 0.75 μM) but only modest AChE inhibition (IC_50_ = 8.2 μM).

The moderate AChE inhibition and weak MAO-B-inhibitory activity of compounds that were generated using linkage or fusion techniques highlights the need for a stiff scaffold to produce effective MAO-B inhibitors. Moreover, research has been conducted on the use of antidepressants such as sertraline and fluoxetine as potential treatments of AD [134]. These compounds were created by combining structural units of antidepressants with tacrine, donepezil, and other antidiabetic medications [136]. Some other compounds had strong and well-balanced dual inhibitory actions; however, the outcomes required further investigation.

## 5. Computational Methods to Design Dual Inhibitors

Computer-aided drug design dramatically enhances the drug development process by accelerating the discovery of potential drug candidates, optimizing their properties, and reducing the time and cost associated with bringing new therapies to market. For example, the selectivity index for MAO-B was significantly increased by a thousand-fold when Gogineni et al. used computational methods to improve the flavonoid acacetin 7-*O*-methyl ether’s selectivity [135]. Similarly, the Wang group evaluated the subtype selectivity of MAO inhibitors using the Three-Dimensional Biologically Relevant Spectrum (BRS-3D), a representative chemical database in CADD [137]. The natural substance kaempferol was discovered to be an AChE inhibitor in a noteworthy study conducted by the Zhou group [72]. Simultaneously, kaempferol was identified by another research team as a selective inhibitor of human MAO-A [138]. The concept of considering different targets in drug discovery was emphasized by this dual identification, which also highlights the adaptability of compounds. Typical in silico methods include ligand-based (such as SAR, machine learning, pharmacophore mapping, and ligand-based virtual screening) and structure-based approaches (such as molecular docking, molecular dynamic simulation, and structure-based virtual screening). A summary of the commonly used computational techniques in drug design is presented in Table 3.

### 5.1. Virtual Screening (VS)

VS has been widely exploited to screen potential hits against multiple targets for the treatment of AD. In an attempt to identify dual inhibitors for AChE and MAO-B from a large compound library, Kumar et al. [139] employed both structure-based and ligand-based VS techniques to maximize the chances of finding effective compounds. Khan et al. conducted a VS study focusing on identifying novel compounds capable of targeting both AChE and MAO-B [140]. They utilized an extensive compound library and applied advanced VS algorithms to predict the binding affinities and selectivity of the compounds. The study employed a multi-step screening process, starting with a broad screening of the library followed by more focused, high-precision docking simulations to refine the selection.

The VS approach successfully identified several promising S-alkyl phthalimide- and S-benzyl-oxadiazole-quinoline hybrids with potential inhibitory activity against MAO and AChE. These selected compounds were then subjected to further experimental validation to confirm their inhibitory effects [140]. In another study, Salman et al. utilized VS to find dual inhibitors for AChE and MAO-B [141]. Their study focused on a diverse set of chemical libraries, employing a combination of high-throughput VS and detailed molecular docking studies. The screening process identified multiple promising compounds that were further optimized through structure–activity relationship (SAR) studies. The optimized compounds demonstrated significant inhibitory activity against both enzymes in experimental assays, confirming the potential of VS in identifying effective dual inhibitors. Likewise, James et al. [142]. exploited virtual screening to investigate the potential interaction of a series of twenty-four phytoconstituents with MAO-B and AChE linked to neurodegenerative diseases. In their study, piperine and rutin stood out for their higher inhibitory activity against these enzymes, suggesting that they might have neuroprotective properties [142].

### 5.2. Other Molecular Modeling Methods

**Figure 20:**
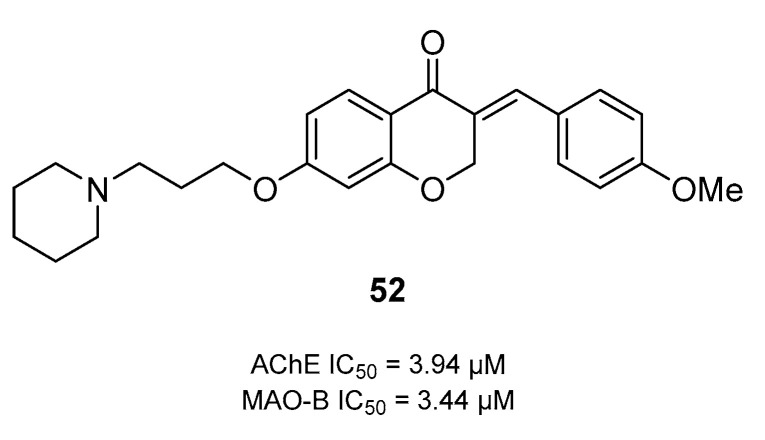
Structure of a balanced dual inhibitor **52** based on chromone-4-one.

Molecular docking has been a valuable tool in most of the research conducted to design and develop dual AChE/MAO-B inhibitors in the treatment of AD. By simulating the interactions, highlighting important substitutions and how the molecule fits into the enzyme binding pockets, researchers have exploited molecular docking extensively in the process of using dual inhibitors against AChE and MAO-B, as discussed in the previous chapter. Recently, a drug reprofiling study was conducted by Mateev et al. [143] to evaluate the potential of FDA-approved drugs as dual AChE and MAO-B inhibitors in the treatment of Alzheimer’s disease. A consensus molecular docking approach was employed using GOLD 5.3 and Glide software for the assessment and ranking of acquired poses. As a result, four top-ranked drugs, namely, rebamipide, diflunisal, dolutegravir, and loracarbef, were reported to have dual activity. Overall, the in vitro experiments validated the in silico findings; however, the MAO-B assay obtained two false positives, while rebamipide, diflunisal, and dolutegravir showed inhibition of AChE. Only one drug, dolutegravir, an antiretroviral agent, displayed strong dual inhibition potential, i.e., 68% inhibition of 10 AChE (10 μM) and 41% inhibition of MAO-B (1 μM). The interaction profile from the molecular docking analysis of dolutegravir revealed that it was present near the peripheral active site (PAS) instead of the binding pocket of AChE. Important binding residues of PAS (including Tyr34, Tyr72, Asp74, Tyr124, and Trp 286) were seen to be involved in the binding of dolutegravir. Moreover, five hydrogen bonds were noticed in the formation of the stable dolutegravir–AChE complex. In addition, an active water molecule (HOH953) actively formed two hydrogen bonds with the carbonyl moieties. In another study, Wang et al. conducted the comprehensive design and synthesis of homoisoflavonoids derivatives to inhibit both AChE and MAO enzymes simultaneously to treat AD [144]. Compound **52** (Figure 20) displayed a balanced dual inhibition such that IC_50_ = 3.94 µM with AChE and IC_50_ = 3.44 µM for MAO-B. To explore the binding mode of compound **52** in the binding pockets of AChE and MAO-B, the molecular docking approach was employed using the Discovery Studio software. Simulation results revealed that compound **52** was positioned near the PAS and interacted with both the PAS and CAS of the AChE enzyme. Trp84, Tyr121, Ser122, Trp279, Phe330, Phe331, and Tyr334 were the primary interacting residues in the AChE pocket. Moreover, the benzyl group of compound **52** showed proper positioning on the cofactor FAD through the Tyr 435 and Tyr398 residues in the pocket of MAO-B. Other important interacting residues were also highlighted, including Pro102, Pro104, Leu167, Leu164, Trp119, Ile316, and Phe186 [145].

**Figure 21:**
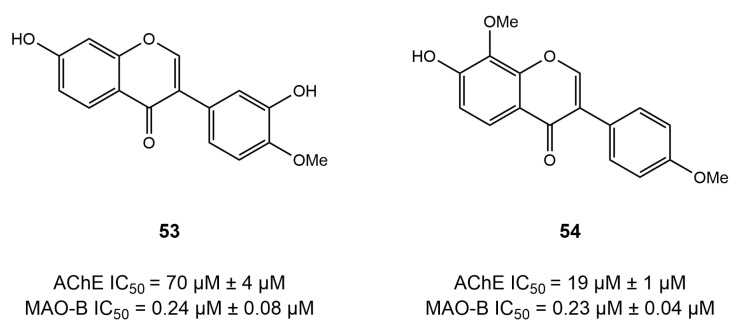
Selective inhibitors of the MAO-B enzyme based on chromen-4-one [146].

In an interesting study, Oh et al. performed docking studies on herbal compounds from *Maackia amurensis* to evaluate its neuroprotective effect and potential to inhibit AChE and MAO-B [145]. The stem of Maackia amurensis, commonly known as Amur Maackia, has previously been used in the treatment of cancer. In this study, they screened 19 compounds (13 isoflavones) from the *Maackia amurensis* herbal library using SAR and further employed a molecular docking simulation to check their activity in the binding pocket of hMAO-B and AChE. Only compound 13-a, prenylflavonoid (isolupalbigenin), showed effective dual inhibition of both AChE and hMAO-B; i.e., IC_50_ = 20.6 and 4.83 μM, respectively. They also discovered that compounds **53** and **54** (calycosin and 8-*O*-methylretusin) are reversible and selective inhibitors of MAO-B enzyme (Figure 21).

The docking studies revealed that both calycosin and 8-*O*-methylretusin fit efficiently into the active site of MAO-B; however, calycosin was observed to form a hydrogen bond with Cys172, which was absent in the case of 8-*O*-methylretusin [145]. Similarly, the molecular docking method was utilized by Sang et al. [101], while developing chalcone- and donepezil-based compounds to achieve improved AChE/MAO-B inhibition potential. Simulation results discovered the positioning of ligands in the binding pockets of both receptors, in addition to crucial interacting residues (refer to the dual inhibitors chapter). Moreover, oxime ether-containing drugs possess dual inhibitory potential and have been extensively assessed in the treatment of AD. When designing and synthesizing a series of chalcone oxime ethers, Oh et al. [106] utilized a molecular docking approach to obtain an insight into the binding pattern of oxime ethers into the pockets of AChE and MAO-B. The results showed that chalcone ether oxime fairly interacted with the FAD via Tyr398 and Tyr435 and established π–π interactions with Tyr326 residue. In addition, the role of molecular docking studies has been impeccable in exploring dual function inhibition of coumarins in the treatment of AD. Aspiring to unfold the binding pattern of the coumarin core into the pockets of AChE and MAO-B, numerous studies [111,112,114] have employed molecular docking. The results of molecular docking studies revealed that coumarins correctly occupied the active site of AChE and interacted with key residues, such as Trp86, Gly121, Trp286, Tyr341, Gly448, and His 447. Similarly, the coumarin core was discovered to occupy the substrate cavity of MAO-B, where important residues were observed to establish hydrogen bonds and hydrophobic interactions with Tyr188 and Phe343, Tyr60, Tyr398, and Tyr435, respectively.

**Figure 22:**
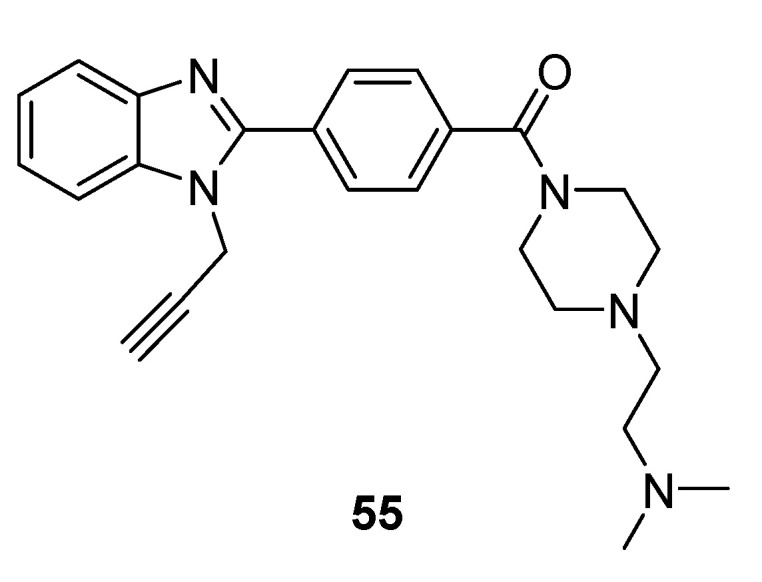
Structure of benzimidazole **55** studied in complex with AChE [83].

In addition to docking simulations, molecular dynamic (MD) simulations provide an insight into the binding affinities and the conformational stability of ligands in a complex with the protein of interest. MD simulations offer a deeper understanding of the molecular basis of inhibition and structural changes over time, guiding the design and development of more effective treatments for AD. Osmaniye et al. [83] validated the stability of AChE-compound **55** complex (Figure 22) through molecular dynamic (MD) simulations while improving the dual activity potential of benzimidazole derivatives (Figure 19). The results showed an RMSD value of 1.25 Å for AChE, demonstrating the compactness of the system during simulations. Interestingly, the region between the 283 to 324 amino acid residues was observed to be stable because of the interactions generated by compound **55** in the AChE pocket. The inhibitor–enzyme complex remained stable throughout the simulation. Moreover, hydrogen bonding between Asp74, Glu202, Tyr341, Tyr124, Phe338, Trp286, and His447 and compound **55** were found to be preserved during the simulations.

In recent years, drug discovery approaches have been dramatically elevated with the advancements in machine learning (ML) algorithms. ML approach facilitates the discovery of effective and novel multi-target drug candidates by delineating the complex relationships between molecular features/descriptors and the biological activity. Although many researchers have utilized ML techniques to develop inhibitors against one receptor, few studies have reported the application of ML techniques to develop dual AChE/MAO-B inhibitors. Most recently, Hou et al. [146] developed six classification models, including support vector machines (SVM), k-nearest neighbor (KNN), light gradient boosting machines (LGBM), random forest (RF), gradient-boosted decision tree (GBDT), and deep neural network (DNN), by collecting the inhibition dataset against AChE and MAO-B from the literature and the CHEMbL database. They employed DARGON descriptors along with four different fingerprints to train and optimize the machine learning algorithms. Among all, GBDT presented the best performance and optimal statistics (MCC = 0.70; accuracy = 0.89; precision 0.88) for dual AChE/MAO-B inhibition. In addition, important functional groups and descriptors, including halogen atoms, piperidine, and coumarin were highlighted to contribute directly towards dual AChE/MAO-B inhibition. However, the results are yet to acquire in vitro validation for dual inhibition in the treatment of AD. This study demonstrates the potential of ML to streamline the development of dual inhibitors, leveraging large datasets and advanced algorithms. ML techniques can suggest structural modifications and accurately identify potent inhibitors.

## 6. Recent Advances in the Development of Inhibitors for the Treatment of AD

The limited efficacy and failure of conventional AD drugs to halt disease progression impose a significant need to modify AD treatment. Over time, a better understanding of the multifactorial pathophysiology of AD, along with advanced drug design technology, has amended the practices to discover novel AD drugs. The general trend of shifting from one-drug, one-target to one-drug, dual- or multiple-target offers a promising prospect of AD therapeutics [147]. The dual-target inhibition has shifted the paradigm to develop AD drugs as it poses less off-target toxicity, more safety, and prolong synergistic effects.

A dual AChE/MAO-B inhibitor called ladostigil has completed its phase-II trial and been declared to be non-toxic and well-tolerated among the patients [148].

**Figure 23:**
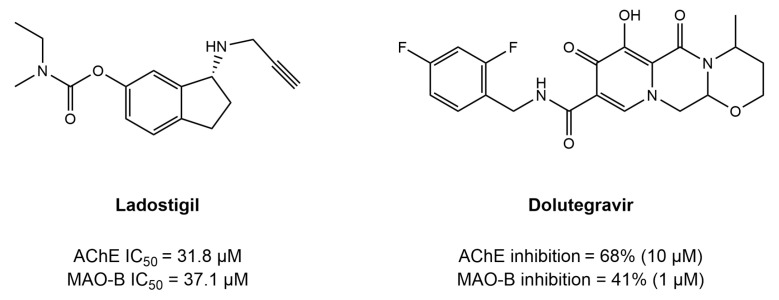
Example of tested inhibitors for the treatment of AD [143].

FDA-approved drugs have been investigated for dual AChE/MAO-B inhibition potential. Consequently, dolutegravir is noteworthy for its strong binding to both enzymes, acceptable safety profiles, and penetration across the blood–brain barrier (Figure 23). Additionally, rebamipide, which is used to treat recurring aphthous ulcers, has shown encouraging characteristics that suggest additional experimental testing [143]. Several other lead compounds, such as coumarin derivatives and donepezil-based derivatives resulting from fusion and hybrid techniques, have presented remarkable dual AChE/MAO-B inhibition potential (Figure 19). Moreover, numerous other dual inhibitors targeting BuChE, GSK-3β, and BACE1 in combination with AChE have been explored and found to significantly delay cognitive decline in addition to their anti-neuroinflammatory and neuroprotective potential [149,150,151]. According to one report [152], there are currently 31 drugs in phase-I, 87 in phase-II, and 36 in phase-III clinical trials in the AD research landscape. It is noteworthy that most drugs that reached phase-II clinical trial target tau protein and neurotransmitter receptors.

A handful of disease-modifying therapies for AD that propose mechanisms to halt the disease progression have emerged over the last decade. An oligosaccharide, sodium oligomannate, was approved in China in 2019 for its conditional use in AD and is currently in phase-IV clinical trials. Despite its controversial mechanism of action and efficacy, it was discovered that GV-971 delays disease progression by inhibiting the aggregation of Aβ fibrils and neuroinflammation. However, the drug is expected to complete clinical trials for safety and efficacy and make its way to the market by the end of 2025 [153]. Moreover, monoclonal antibodies (Aducanumab, donanemab, and lecanemab) have demonstrated disease-modifying outcomes in clinical trials. The antibodies delay cognitive decline by targeting Aβ plaques and inhibiting Aβ aggregation; however, they impose a great risk of ARIA. Aducanumab has already been approved by the FDA, and donanemab successfully completed its clinical trials and is in the process of being introduced into the market [63]. Additionally, combination therapies, such as a cholinesterase inhibitor with an antioxidant or a metal chelator, or the combined use of more than one cholinesterase inhibitor, might provide desirable effects in AD research. However, combination therapies present certain challenges, particularly with respect to off-target toxicity and efficacy, which should not be neglected. Currently, the goal of AD research is to create therapies that alter or reverse the condition. One such strategy is gene therapy, which makes use of the ability to modify genetic components linked to AD. Targeting the pathogenic mechanisms underlying AD, lipid nanoparticles, and dendrimer-based treatments have emerged as promising approaches to improving drug delivery efficiency. These cutting-edge tactics represent a turn away from treating AD symptoms and towards tackling the disease’s underlying causes, which is an important first step towards creating more thorough and potent therapies [68].

Furthermore, novel approaches to drug delivery are being investigated to enhance the brain-penetration of substances and to overcome problems associated with the blood–brain barrier. As the field of AD therapy is constantly evolving, the development of dual AChE/MAO-B inhibitors represents a promising strategy by which to address its complex pathology. Coupled with computational and AI-driven drug design techniques, these approaches hold the potential to optimize therapeutic outcomes for more effective AD treatments in the future. Overall, AI has been exploited in diagnosis, biomarker prediction, drug design, efforts to understand the mechanism of action, clinical trials, and prognosis, and holds promise for advanced treatment of AD over a period of time.

## 7. Conclusions and Prospect

The multifactorial nature of AD continues to challenge conventional drug development paradigms. This review has highlighted the increasing relevance of multi-target drug design strategies aimed at simultaneously modulating several pathological pathways implicated in AD.

Despite being the most promising target in the pathology of Alzheimer’s disease, the AChE drugs approved by the FDA, namely, rivastigmine, donepezil, and galantamine, only provide symptomatic treatment. MAO-B has emerged as another critical enzyme against AD due to its overexpression and consequent oxidative stress in the brain of AD patients. Lately, the multi-target drug concept has shifted the dynamics of AD drug design, given the complex multifactorial nature of the disease. The dual function inhibition approach of AChE and MAO-B could provide synergistic effects by enhancing the neuroprotective ability and memory simultaneously. This review underscores the evolving landscape of AD treatment, particularly highlighting the promise of MTDLs that have been found to concurrently inhibit both MAO-B and AChE in recent years.

Generally, dual inhibitors that target both AChE and MAO-B are categorized as chalcones, chromones, coumarins, imine, and hydrazone scaffolds on the basis of their structural information. Recent advances in in silico methodologies such as pharmacophore modeling, molecular docking, molecular dynamics simulations, and machine learning have greatly facilitated the rational design of such multi-target compounds. The integration of ligand-based and structure-based approaches, particularly when supported by high-resolution structural data from techniques like Cryo-EM, offers a powerful toolkit for identifying and optimizing compounds with polypharmacological profiles. In light of those facts, we provide an in-depth study of the methods employed to develop dual inhibitors. SAR and molecular modeling present a significant stride towards effective multi-target therapies for AD. It was discovered that Tyr121, Phe295, and Phe288 generated hydrogen bonding, whereas Trp289, Phe330, Trp279, Phe334, Phe338, and Tyr341 were involved in π–π interactions in the pocket of AChE. However, Tyr334 and Tyr72 were observed to form hydrogen-bonding and π–π interactions with the ligands. In case of MAO-B, Arg42, Pro102, Cys172, Tyr188, Ile198, Ile199, Gln206, Tyr326, Tyr398, and Tyr435 were the prominent interacting residues. These residues played a critical role in predicting the activity potential of dual inhibitors for AChE and MAO-B. Docking simulations have facilitated a deeper understanding of enzyme active sites and binding interactions, leading to the identification of promising inhibitors.

Despite recent significant advancements, the development of effective dual inhibitors targeting AChE and MAO-B in the treatment of Alzheimer’s disease remains challenging. One big challenge is the structural difference of both enzymes; the active site of AChE is present in the depth of a deep gorge, whereas MAO-B has its active site at the entrance, necessitating the design of dual inhibitors that possess desirable pharmacology with balanced activity strenuousness. Furthermore, in spite of promising computational and preclinical data, many of these approaches remain to be validated in clinical settings. Future research should focus on experimental confirmation of in silico predictions, improved models of blood–brain barrier penetration, and acquiring a deeper understanding of how target engagement translates to functional and cognitive outcomes in vivo.

In conclusion, multi-target drug design, guided by advanced computational tools and informed by structural biology, represents a promising frontier in AD research. The continued integration of these approaches with experimental validation will be essential to translate these strategies into effective and disease-modifying therapies.

## Figures and Tables

**Table 1:** Key molecular players, therapeutic targets, and potential gene functions associated with various signaling pathways implicated in Alzheimer’s pathology.

Molecular Signaling Pathways	Therapeutic Targets	Gene Function
**Amyloid Cascade Signaling**	Aβ, APP, BACE1, PSEN1, PSEN2, ApoE	Amyloid precursor protein (APP)—synthesis of Aβ peptides, implicated in plaque formation; BACE1—β-secretase enzyme; PSEN1 and—components of the γ-secretase complex; ApoE—apolipoprotein E involved in Aβ clearance and metabolism
**τ and Neurofibrillary Tangles**	τ protein, kinases	τ protein—microtubule stabilization; kinases—regulate τ phosphorylation in normal conditions; aberrant phosphorylation leads to neurofibrillary tangle formation
**Genetic Mutation**	APP, PSEN1, PSEN2, ApoE4	Variations in APP, PSEN1, PSEN2, and ApoE4 associated with increased risk of Alzheimer’s disease
**Neurotransmitter Signaling**	NMDA receptor, AChE	NMDA receptor—critical for synaptic plasticity; AChE—enzyme involved in acetylcholine breakdown; glutamatergic and GABAergic receptors—essential for neurotransmission
**Mitochondrial Dysfunction**	Mitochondrial respiratory enzymes, mitochondrial ROS	Enzymes involved in mitochondrial respiration and ROS production
**Oxidative Stress**	Reactive oxygen species, mitochondrial dysfunction	ROS—reactive oxygen species; mitochondrial dysfunction—impaired energy production and oxidative stress
**Neuroinflammatory Signaling**	Proinflammatory elements such as chemokines	Various cytokines, chemokines, and reactive species implicated in neuroinflammation
**Ubiquitin–Proteasomal System**	Ubiquitin activating enzyme (E1), ubiquitin conjugating enzyme (E2), UCHL1, 26S proteasome	Components of the ubiquitin–proteasome system involved in protein degradation
**Protein Misfolding and Molecular Chaperones**	Heat shock proteins (HSPs), HSP60 and HSP70	Molecular chaperones (HSPs)—assist in protein folding and preventing misfolding
**Insulin Signaling**	Insulin receptors, insulin degrading enzymes, Akt	Insulin-related proteins involved in glucose metabolism and signaling pathways
**Excitotoxicity**	Glutamate, NMDA receptors	Glutamate receptors—involved in excitatory neurotransmission
**Neurotrophic Factors Signaling**	Brain-derived neurotrophic factor, nerve growth factor, neurotrophin-3 (NT-3), neurotrophin-4/5, Trk receptors	Neurotrophic factors—promote neuronal survival and growth
**Wnt/βCatenin Signaling**	βcatenin, GSK3β	β-catenin—key component in Wnt signaling; GSK-3β—glycogen synthase kinase 3β
**Leptin Signaling**	Leptin, leptin receptor (ObR)	Leptin—hormone involved in energy regulation; ObR—Leptin receptor

**Table 3:** Overview of computational approaches, including virtual screening and molecular modeling techniques, utilized for the design and development of dual AChE/MAO-B inhibitors.

Approach Name	Description	Advantages	Disadvantages
Virtual Screening	In silico screening of large compound libraries to identify potential candidates	Accelerated lead identification	Dependent on accurate databases and models
Molecular Docking	Predicts the preferred orientation of one molecule to a second when bound to form a stable complex	Efficient exploration of ligand–receptor interactions; rapid evaluation of binding affinities	Accuracy depends on force-field parameters and model parameters
Molecular Dynamics	Simulates the motion of atoms and molecules over time, allowing exploration of dynamic behavior	Provides insights into dynamic behavior of biomolecular systems; allows for assessment of structural stability	Computationally intensive; may require significant resources

## Data Availability

Not applicable.

## Abbreviations

The following abbreviations are used in this manuscript:

AD	Alzheimer’s disease
CNS	Central nervous system
AChE	Acetylcholine esterase
EOAD	Early-onset Alzheimer’s disease
LOAD	Late-onset Alzheimer’s disease
ChAT	Choline acetyl transferase
VAChT	Vesicular acetyl choline transporter
NMDA	*N*-methyl-D-aspartate
BACE1	Beta-site APP
RAGE	Receptor for advanced glycation end products
ROS	Reactive oxygen species
PHFs	Paired H=helical filaments
MAO-B	Monoamine oxidase B
APP	Amyloid precursor protein
Aβ	Amyloid-β
ARIA	Amyloid-related imaging abnormalities
NFT	Neurofibrillary tangles
PAS	Peripheral anionic site
BBB	Blood–brain barrier
CAS	Catalytic active site
SAR	Structure–activity relationship
VS	Virtual screening
